# Patterns of psychotropic drug dispensation in Portugal amidst the COVID-19 pandemic and beyond

**DOI:** 10.1038/s41598-026-50987-0

**Published:** 2026-05-20

**Authors:** David Belchior, Luís Madeira, Rui Henriques

**Affiliations:** 1 INESC-ID and Instituto Superior Técnico, Universidade de Lisboa, Lisbon, Portugal; 2 Instituto de Medicina Preventiva, FMUL, Portugal and Hospital CUF Descoberta, Lisbon, Portugal

**Keywords:** Mental health, Psychotropic drugs, Dispensation trends, Public health, Psychopharmacology, Diseases, Health care, Medical research, Psychology, Psychology

## Abstract

The increasing prevalence of mental health disorders has been matched with growing psychotropic drug consumption rates around the world. Assessing psychopharmacoepidemiological trends and their determinants is essential to guide medical care delivery and public health policies. However, nation-wide studies on up-to-date consumption patterns are scarce and generally disregard relevant pharmacological, medical, socio-demographic, and economic covariates. Previous studies on the Portuguese case, known for critically high consumption rates of benzodiazepines and antidepressants, are limited to the COVID-19 pre-pandemic period. This study uses the full (electronic) dispensation registry of antidepressants, benzodiazepines and zolpidem, antipsychotics and mood stabilisers in Portugal during the years of 2019 to 2022 to model relevant prescription and consumption patterns prior to, during and after the COVID-19 pandemic. The data reveal an accelerated growth trend in antidepressant consumption since 2020, with a 7.41% annual increase in dispensed defined daily doses and a growing user base (1.7M users, 16.1% of the population), overtaking benzodiazepines and zolpidem as the class with most active users. The total annual expenditure has increased 14M€ between 2020 and 2022 (nearly 2M€ in public copayment), notwithstanding price drops in diverse antipsychotic drugs.

## Introduction

Population mental health worsened during and after the COVID-19 period, with sustained elevations in depression and anxiety across Europe^1,2^, particularly among women and young people^3^, stressing the need for an actionable monitoring of psychotropic drug consumption patterns. In particular, Portugal is the fourth country in the European Union with highest estimated risk of depression in the adult population (with 61% of the population at risk in 2022), and the second of the member states of the Organisation for Economic Co-operation and Development (OECD) with highest estimated consumption of antidepressants^4^. Portugal further shows a long-standing reliance on benzodiazepines, with one of the highest dispensation rates among OECD countries, largely driven by high community and primary care prescribing, prolonged use in older adults, and limited access to non-pharmacological care^5–7^.

Various cross-generational retrospective studies have been complementarily undertaken to assess psychotropic drug consumption trends in recent years^8–14^. In the case of Portugal, pre-pandemic indicators reveal an annual increase of 9.9% and 11.6% in the consumption of antipsychotics and antidepressants, respectively^7^, with the majority of psychotropic drug prescriptions issued by general practitioners (GPs) and only 21% originating from psychiatric and neurological specialties^5,7^. Notwithstanding, the assessment of dispensing and expenditure trends, and their underlying determinants, has not been extended to more recent years to capture the impacts of the COVID-19 pandemic, as has been reported in other countries such as Croatia^8^, France^9,10^, Spain^11^, Brazil^12^, and Italy^13^.

In this context, this study aims at profiling nation-wide psychopharmacoepidemiological trends in the Portuguese territory between 2019 and 2022 as a function of relevant covariates, including geographic location, user’s demographic profile, pharmacological properties, and prescriber specialty, effectively extending the analysis to the 2020–2022 period. The complete nation-wide dispensation registry on antidepressants, benzodiazepines and zolpidem, antipsychotics, mood stabilisers and pregabalin on the Portuguese Ministry of Health’s *Prescrição Eletrónica Médica* system was used to this end. Access to dispensation patterns offers the opportunity to test pharmacological, medical, demographic and geographic determinants during and after the COVID-19 pandemic period, as a way to guide the design and implementation of public policy actions.

## Methods

For the comprehensive assessment of psychotropic drug dispensation patterns, this study tests trends, determinants, and pandemic-related changes across four major psychotropic classes using annual dispensation rates according to: (i) defined daily doses per 1,000 inhabitants per day (DID); (ii) user base (number of users and population coverage); and (iii) expenditure volumes (state-funded copayments and total expenditure). Dispensation rates are further stratified and standardised by relevant covariates—including sex, age group, dispensing location, prescriber specialty, active ingredient, and drug formulation—to test determinants. Statistical analyses include stratified trend analyses, hypothesis testing, regression modeling, and interrupted time series analysis.

### Data collection

This study presents a nationwide retrospective analysis based on the complete dispensation registry of Portuguese citizens aged 18 or older, as recorded in the Portuguese Ministry of Health’s *Prescrição Eletrónica Médica* (PEM) system between January 1, 2019, and December 31, 2022, on psychotropic drugs approved for commercial use by Infarmed. The full list of drugs considered, divided by class and subclass, including their anatomical therapeutic chemical (ATC) classification code (if existent) and defined daily dose by route of administration (DDD), as well as their various commercialized packages, can be found in the supplementary material (Table S1). Following current conventions, drug consumption incidence was quantified in DDD per 1000 inhabitants per day (DID), a metric often used as a standardised, drug-independent reference proxy for incidence on a population’s consumption patterns^7,15^.

The drug categorisation is aligned with the ATC classification system, albeit with some exceptions. Benzodiazepines and zolpidem are grouped into a single category, regardless of their additional classification as anxiolytics or sedatives/hypnotics; this includes the combination of chlordiazepoxide with clinidium bromide, an antispasmoid, with the placed DDD matching chlordiazepoxide’s as a standalone drug. Pirlindole has no ATC code as of yet, and thus a DDD assignment of 200 mg/day was established based on previous studies with the same active ingredient^16,17^. As valproic acid and lamotrigine (classified as antiepileptics) are also administered as mood stabilisers, they are included in this class. Pregabalin was also analysed, albeit individually (Appendix C).

In order to standardise demographic deviations across the Portuguese territory, annual data on Portugal’s administrative divisions (municipalities and districts) and their population was retrieved from *Instituto Nacional de Estatística* (INE), Portugal’s statistics institute^18^.

*Ethical and regulatory compliance*. Ethical approval for the target study, including access to and processing of the national dispensation registry, was granted by the Ethics Committee of the Centro Académico de Medicina de Lisboa (CAML) (ref. 340/20). All methods comply with applicable ethical and regulatory standards, including the principles set forth in the Declaration of Helsinki.

### Data processing

The data warehousing process, necessary for the efficient retrieval of dispensation patterns from the large-scale registry of electronic prescription and dispensation acts (+10M yearly records), entails a processing stage, where the raw data are cleaned and harmonised between the different sources (prescriptions, drugs, packaging, medical specialties, and demographics) before their consolidation. The system developed for the extraction, transformation and loading of the data, as well as its aggregation into the target statistics, is divided into three major layers: a PostgreSQL relational database; a Django back-end responsible for processing the raw data, as well as their manipulation and provisioning within a dedicated interface that communicates with the database; and a Python front-end for the creation of data requests and the conversion of their results into dataframes, which are then plotted using Matplotlib. The end-to-end software is available on GitHub (https://github.com/ruihenriques/bpsi.git).

Each dispensation act includes the corresponding timestamp, location (municipality), complete package information, total price and governmental copayment, prescriber’s specialty, and user’s gender and age group. PEM data accounts for 7 age groups, spanning 10 years each, with the exception of the first (18–29) and last (80 or older) groups. In contrast, the yearly (estimated) population statistics retrieved from INE’s census data follow a different organisation, divided in 18 age groups, spanning 5 years each up to the final group, which includes people aged 85 or older, thus requiring modifications to align both sources. First, the 15–19 age group is divided into the 15–17 and the 18–19 groups by considering yearly uniform birth statistics, meaning that the 18–19 group contains 40% of the initial count (rounded up), which is added to the 20–24 and 25–29 groups, creating the 18–29 group. 80–84 and 85 or older groups are merged in a single 80+ age group. Finally, the remaining age groups are presented in 10-year segments.

The location of dispensations is used as a proxy for region-conditional analysis from 2020 onwards. As the data for 2019 are sourced under a different protocol, the registered locations correspond to prescription acts, which are used as a proxy for dispensation acts at pharmacies.

Data from zolpidem, cariprazine and clozapine only feature in the supplied data since 2020, despite their previous approval by Infarmed. In this context, to provide a more statistically accurate estimate on dispensed DID and overall costs on their respective classes, the dispensation of these specific drugs was linearly extrapolated for the year of 2019.

Region-specific statistics related to dispensed DID are standardised by the national distribution of demographic sections using the *k* coefficient,

$$\begin{aligned} k = \frac{Population(Region) \times Population(Portugal, Age Group, Gender)}{Population(Region, Age Group, Gender) \times Population(Portugal)}. \end{aligned}$$

In medical specialty-related analyses, all prescriptions made by practitioners with registered specialties other than Psychiatry and General Practice are grouped into *Other Specialties*. The full list of specialties can be found in Appendix E.

### Statistical analysis

A stratified pattern analysis is conducted using multiple measurement systems – dispensed DID (primary), total and copayment expenditures, and the number of prescriptions and users with active dispensations – against several covariates: pharmacological (class and active ingredient, Appendix E), geographic (district and municipality), demographic (sex and age group), and medical specialty.

Trends are evaluated using a Wald test with a t-distribution at a 5% significance, under a two-sided hypothesis, with the null hypothesis that the mean year-on-year rate of change equals zero.

Statistical testing of changes in dispensation DID across periods is undertaken using the Wilcoxon signed-rank test on estimates obtained from paired age groups (one-tailed testing for statistically significant increases) under two scenarios: average DID from pre-pandemic and pandemic periods, and yearly changes along the pandemic period. For the assessment of periods of accelerated growth in psychotropic drug dispensation, the analysis of year-to-year DID ratios is complemented by an interrupted time series (ITS) analysis. Increases before the pandemic (2018–2019) and after the pandemic onset (2021–2022), with 2020 being defined as the intervention time, are tested via ordinary least squares (OLS) regression using estimates obtained from paired age groups.

## Results

**Fig. 1:**
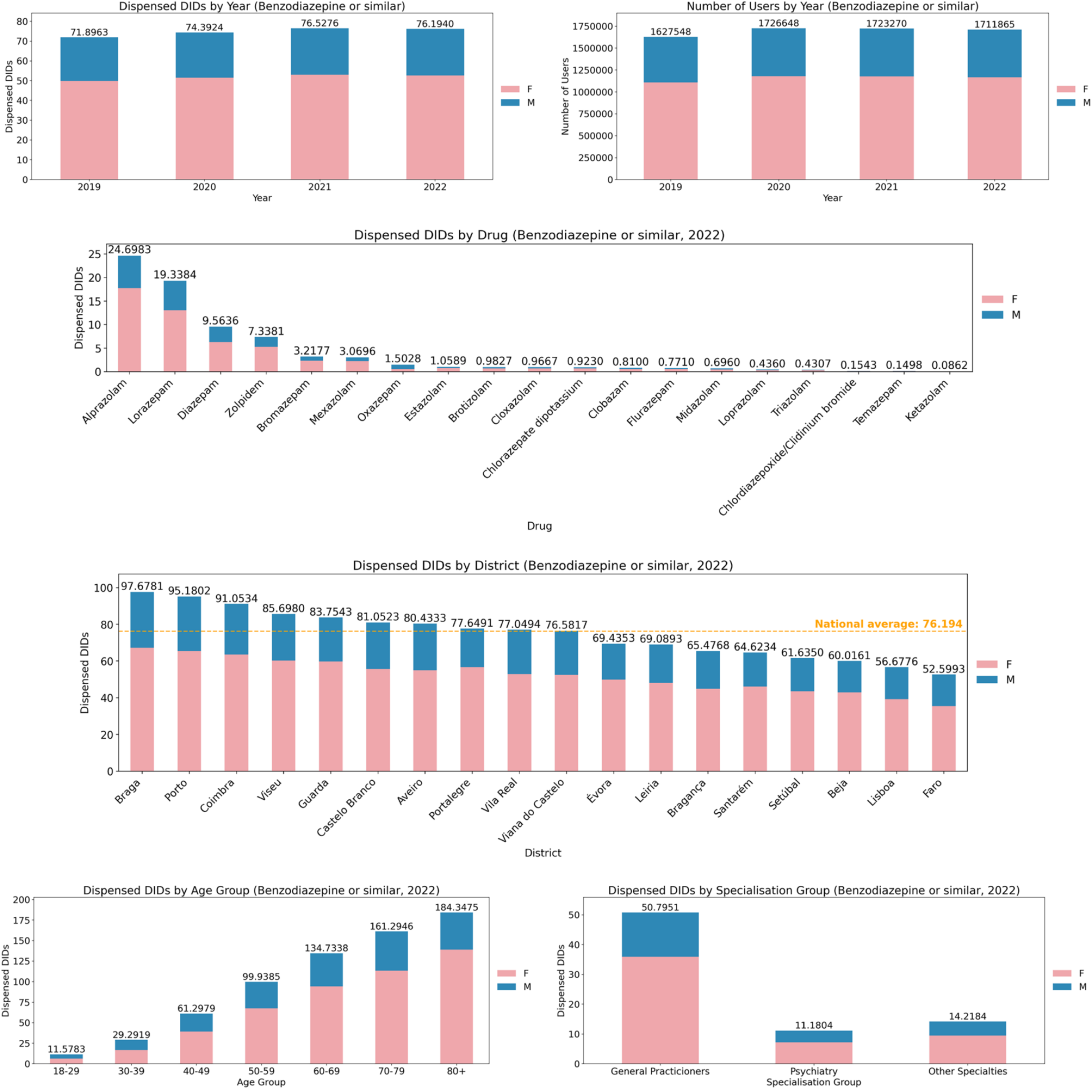
Benzodiazepine and zolpidem dispensation statistics (DID and user count) across districts, demographic sectors and medical specialties (gender is presented using stacked representations).

**Fig. 2:**
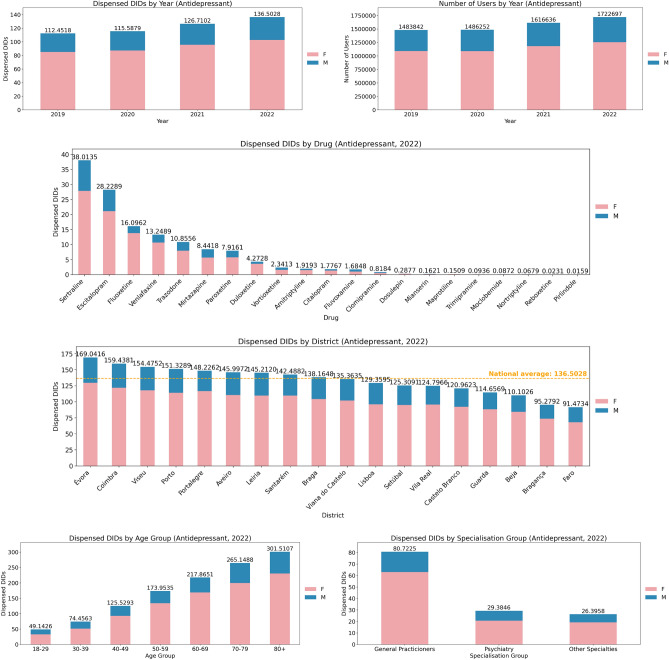
Antidepressant dispensation statistics (DID and user count) across districts, demographic sectors and medical specialties (gender is presented using stacked representations).

**Fig. 3:**
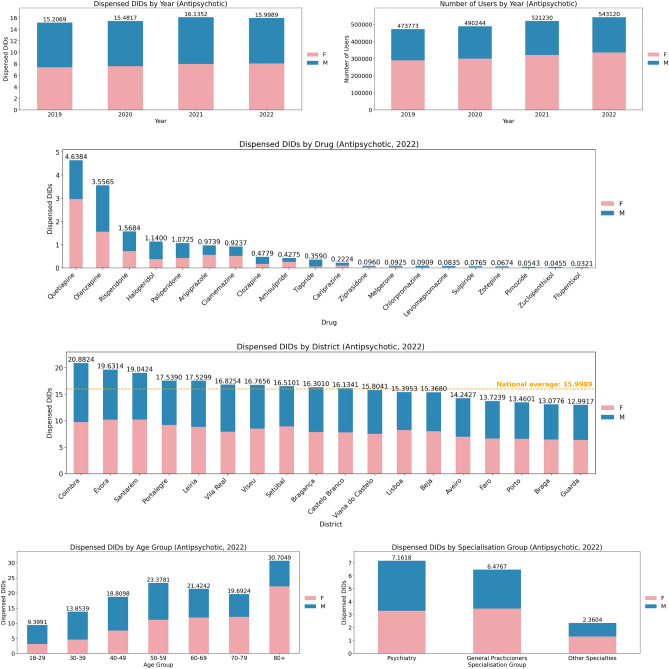
Antipsychotic dispensation statistics (DID and user count) across districts, demographic sectors and medical specialties (gender is presented using stacked representations).

**Fig. 4:**
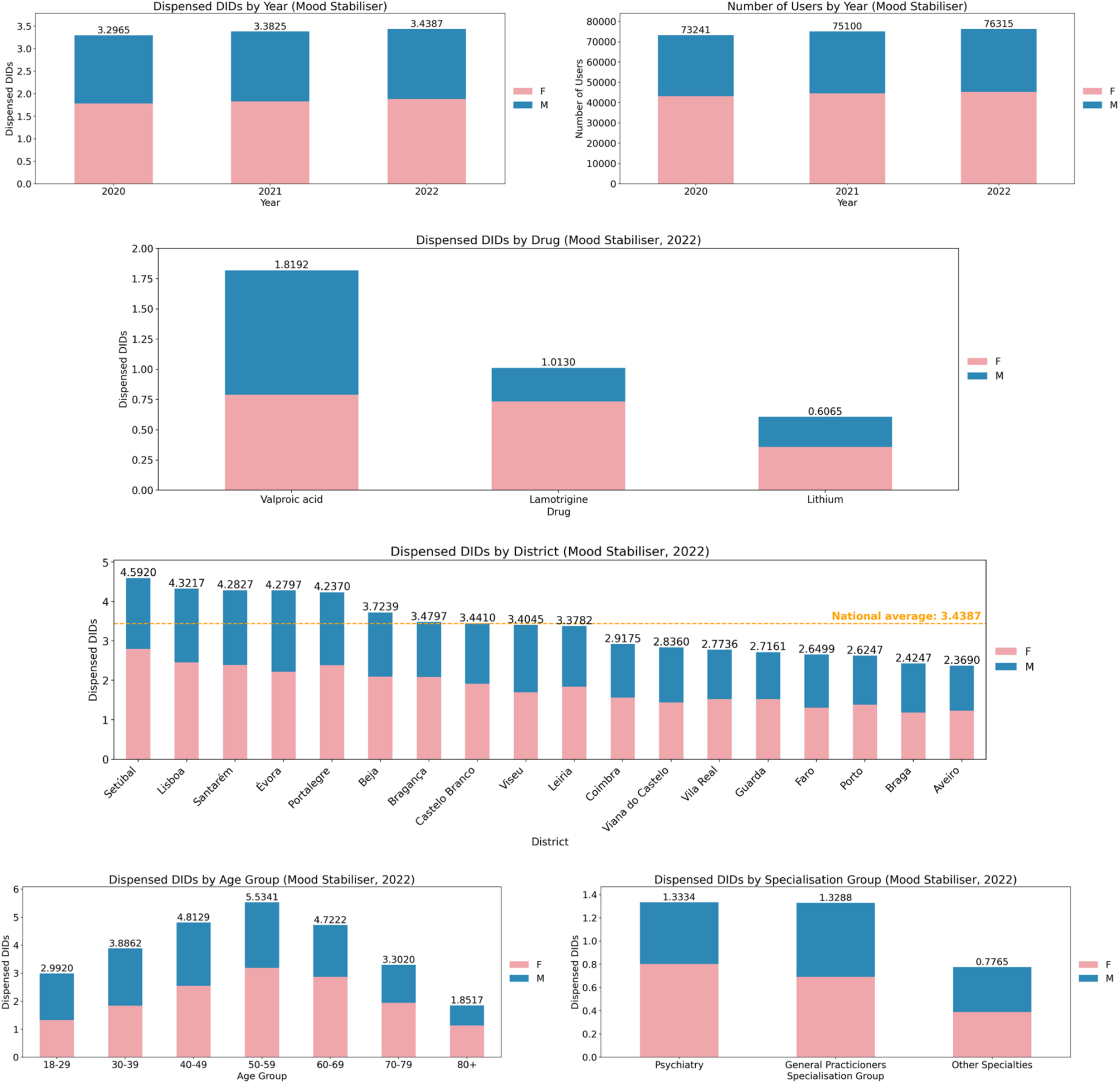
Mood stabiliser dispensation statistics (DID and user count) across districts, demographic sectors and medical specialties (gender is presented using stacked representations).

Key results, organised by psychopharmacological class, can be found in Tables 1, 2, 3 and 4 and Figs. 1, 2, 3 and 4.

### Benzodiazepines and zolpidem

Both the number of users with dispensed benzodiazepines (Fig. 1) and the associated dispensations in DID (Table 1) have remained fairly stable throughout the monitored period, plateauing around 1.7M users—over 15% of the Portuguese population. When looking at individual demographic groups, we can find statistically significant growth trends, mainly driven by the male demographic, the 70–79 age group, and, in particular, the 18–29 age group, where we can find annual DID growth trends in females and males of 9.59% and 7.47%, respectively, since 2019. A differential analysis of the pandemic and pre-pandemic periods, presented in Appendix D, confirms the stabilisation trend, with most age groups exhibiting reduced growth rates or outright declines.

In a drug-centered stance, the prescription of mexazolam has continued the growth trend shown since 2016, while bromazepam, on the contrary, has faced a significant decrease.

**Fig. 5:**
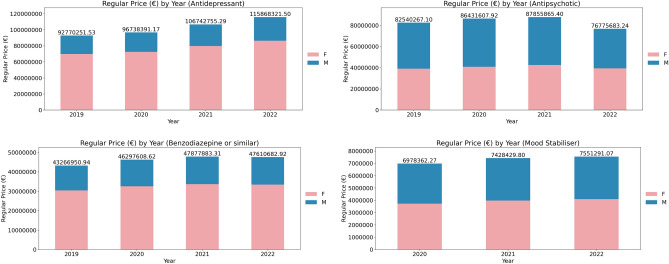
Evolution of the total expenditure of each considered psychotropic drug class between 2019 (or 2020, if unavailable) and 2022.

**Fig. 6:**
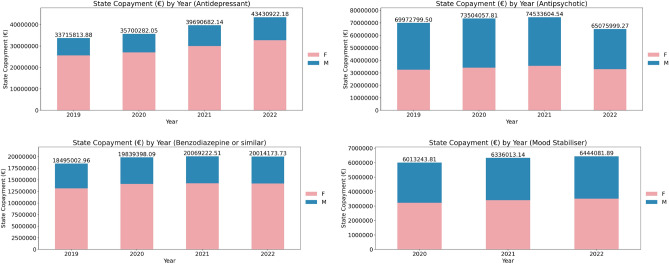
Evolution of the total state-funded copayment of each considered psychotropic drug class between 2019 (or 2020, if unavailable) and 2022.

**Fig. 7:**
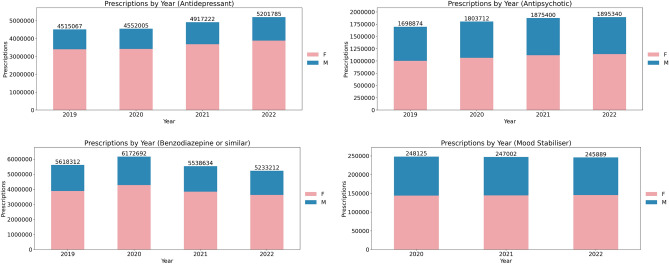
Evolution of the number of prescriptions with at least one dispensation act of each considered psychotropic drug class between 2019 (or 2020, if unavailable) and 2022.

The volume of associated expenditures shows a soft increase since 2020 (Appendix A, Figs. 5 and 6), following the reference inflation, and accompanied by a soft decline in the number of prescription acts (Appendix B, Fig. 7), each showing a higher average number of DID.

### Antidepressants

Results show a marked increase in antidepressant use since 2016 (Wald statistic, Table 6), both in DID dispensation (Table 2), user base (Fig. 2), and associated expenditures (Appendix A). Antidepressants have overtaken benzodiazepines to become the most widely dispensed class of psychiatric drugs by number of users, reaching over 1.7M individuals.

**Fig. 8:**
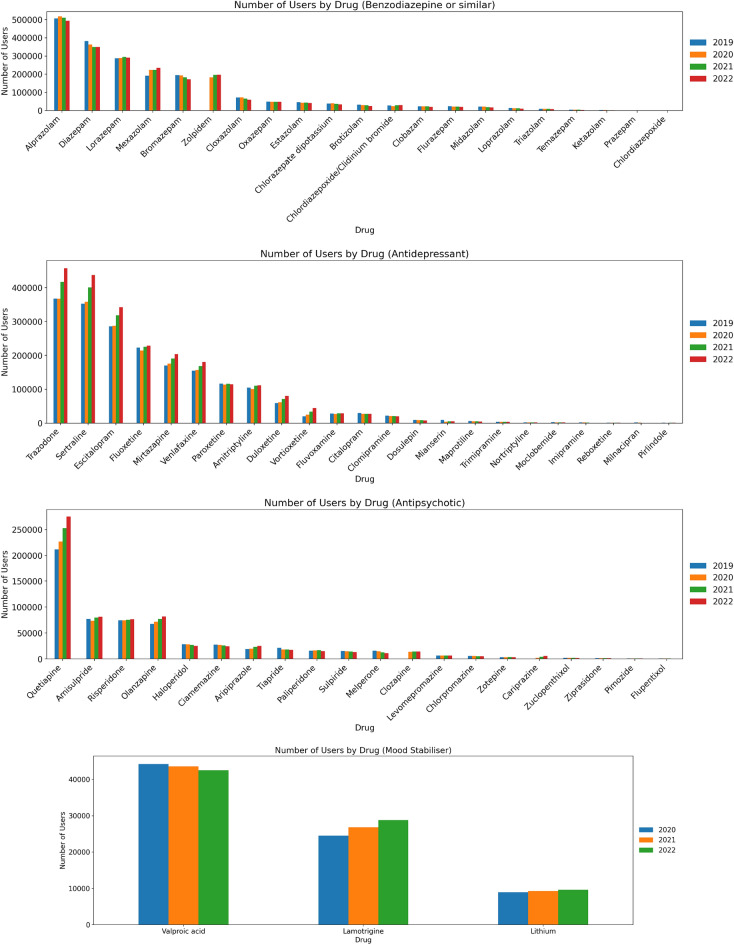
Number of users with at least one dispensation per psychotropic drug, grouped by their respective class, between 2019 (2020 for mood stabilisers) and 2022.

This trend is reflected in the majority of the considered active ingredients (Appendix B, Fig. 8) and in all demographic classes (Table 2), with emphasis on the 18–29 age group, where the average yearly growth relative to the 2019 baseline exceeds 15% in both males and females and has accelerated since the pandemic period (Appendix D).

The generalized upward trend has moderately accelerated during the pandemic period (2021–2022), as confirmed by an interrupted time series analysis targeting 2020 as the intervention year (Appendix D).

### Antipsychotics

An increase in the use of antipsychotics is observable since the pre-pandemic period (Table 1). Notwithstanding, we note that the increase in antipsychotic dispensation since the pandemic onset occurred at a slower rate than in the pre-pandemic period, as evidenced by differential (Table 6) and interrupted time series analysis (Table 8).

**Table 1:** Evolution of dispensed DID in benzodiazepines and zolpidem between 2019 and 2022.

Gender	Age group	2019	2020	2021	2022	Mean change per year, fraction of baseline	*p*-value	95% confidence interval	$r^2$
F	18–29	0.6370	0.7056	0.7841	0.8145	0.0959	0.0140	[0.0466, 0.1452]	0.9722
	30–39	1.8861	2.0130	1.9954	1.9352	0.0069	0.7118	[− 0.0625, 0.0763]	0.0830
	40–49	5.5923	5.9108	5.9941	5.8082	0.0131	0.4563	[− 0.0483, 0.0745]	0.2956
	50–59	9.3648	9.7367	9.9477	9.8139	0.0166	0.1942	[− 0.0206, 0.0538]	0.6492
	60–69	11.6877	12.1447	12.5675	12.5468	0.0257	0.0656	[− 0.0041, 0.0555]	0.8731
	70–79	11.2125	11.4917	11.9762	12.0590	0.0270	0.0294	[0.0067, 0.0473]	0.9422
	80+	9.4550	9.5568	9.7701	9.6668	0.0090	0.1959	[− 0.0112, 0.0292]	0.6466
	**Total**	49.8355	51.5593	53.0351	52.6444	0.0199	0.1072	[− 0.0106, 0.0504]	0.7970
M	18–29	0.5263	0.5738	0.6132	0.6442	0.0747	0.0044	[0.0534, 0.0960]	0.9913
	30–39	1.3444	1.4144	1.4260	1.3996	0.0132	0.3663	[− 0.0358, 0.0622]	0.4016
	40–49	3.0552	3.1927	3.2644	3.2277	0.0193	0.1673	[− 0.0197, 0.0583]	0.6935
	50–59	4.2531	4.4622	4.6425	4.6567	0.0327	0.0499	[0.0000, 0.0654]	0.9027
	60–69	5.0707	5.2153	5.3490	5.3999	0.0221	0.0190	[0.0088, 0.0354]	0.9625
	70–79	4.7381	4.8671	5.0427	5.0771	0.0252	0.0270	[0.0070, 0.0434]	0.9468
	80+	3.0731	3.1077	3.1548	3.1443	0.0085	0.0954	[− 0.0037, 0.0207]	0.8183
	Total	22.0608	22.8332	23.4925	23.5496	0.0232	0.0493	[0.0001, 0.0463]	0.9038
Total		71.8963	74.3924	76.5276	76.1940	0.0209	0.0860	[− 0.0073, 0.0491]	0.8355

**Table 2:** Evolution of dispensed DID in antidepressants between 2019 and 2022.

Gender	Age group	2019	2020	2021	2022	Mean change per year, fraction of baseline	*p*-value	95% confidence interval	$r^2$
F	18–29	2.4246	2.6526	3.4371	4.1304	0.2434	0.0209	[0.0896, 0.3972]	0.9587
	30–39	4.8420	4.9132	5.4363	5.8719	0.0746	0.0347	[0.0132, 0.1360]	0.9318
	40–49	11.7311	11.8981	12.9969	13.7173	0.0602	0.0311	[0.0135, 0.1069]	0.9388
	50–59	16.5712	16.8355	18.2102	19.4443	0.0603	0.0300	[0.0143, 0.1063]	0.9410
	60–69	18.8879	19.4357	21.1572	22.5189	0.0668	0.0173	[0.0285, 0.1051]	0.9656
	70–79	17.4821	17.8966	19.5681	21.1916	0.0732	0.0264	[0.0210, 0.1254]	0.9479
	80+	13.2817	13.8007	14.9794	16.0323	0.0710	0.0113	[0.0383, 0.1037]	0.9775
	**Total**	85.2206	87.4324	95.7853	102.9067	0.0721	0.0224	[0.0249, 0.1193]	0.9557
M	18–29	1.3469	1.4517	1.7897	2.0610	0.1841	0.0181	[0.0761, 0.2921]	0.9642
	30–39	2.0540	2.1221	2.3601	2.6047	0.0920	0.0246	[0.0287, 0.1553]	0.9514
	40–49	3.8631	4.0468	4.4655	4.7870	0.0826	0.0099	[0.0469, 0.1183]	0.9802
	50–59	4.5603	4.7553	5.2963	5.7434	0.0895	0.0153	[0.0414, 0.1376]	0.9697
	60–69	5.5651	5.6593	6.0555	6.5010	0.0576	0.0316	[0.0125, 0.1027]	0.9378
	70–79	5.7239	5.8869	6.4159	6.9781	0.0750	0.0238	[0.0244, 0.1256]	0.9530
	80+	4.1153	4.2334	4.5418	4.9208	0.0662	0.0231	[0.0222, 0.1102]	0.9544
	Total	27.2312	28.1555	30.9249	33.5961	0.0803	0.0193	[0.0316, 0.1290]	0.9617
Total		112.4518	115.5879	126.7102	136.5028	0.0741	0.0215	[0.0266, 0.1216]	0.9574

**Table 3:** Evolution of dispensed DID in antipsychotics between 2019 and 2022.

Gender	Age group	2019	2020	2021	2022	Mean change per year, fraction of baseline	*p*-value	Confidence interval	$r^2$
F	18–29	0.3225	0.3394	0.3838	0.3958	0.0820	0.0256	[0.0245, 0.1395]	0.9494
	30–39	0.5329	0.5439	0.5494	0.5278	− 0.0018	0.8719	[− 0.0451, 0.0415]	0.0164
	40–49	1.1481	1.1652	1.1667	1.1221	− 0.0066	0.5251	[− 0.0440, 0.0308]	0.2255
	50–59	1.5208	1.5618	1.6316	1.6156	0.0233	0.0999	[− 0.0110, 0.0576]	0.8101
	60–69	1.3898	1.4403	1.5387	1.5850	0.0492	0.0106	[0.0272, 0.0712]	0.9790
	70–79	1.1307	1.1536	1.2444	1.2900	0.0503	0.0229	[0.0170, 0.0836]	0.9547
	80+	1.3914	1.4137	1.5007	1.5466	0.0397	0.0222	[0.0138, 0.0656]	0.9562
	**Total**	7.4361	7.6179	8.0154	8.0831	0.0314	0.0316	[0.0068, 0.0560]	0.9378
M	18–29	0.7985	0.8240	0.8282	0.7884	− 0.0033	0.8255	[− 0.0597, 0.0531]	0.0305
	30–39	1.1525	1.1534	1.1432	1.0494	− 0.0277	0.1812	[− 0.0868, 0.0314]	0.6705
	40–49	1.7280	1.7364	1.7596	1.6506	− 0.0121	0.4295	[− 0.0650, 0.0408]	0.3255
	50–59	1.6721	1.7176	1.8162	1.7694	0.0234	0.1934	[− 0.0287, 0.0755]	0.6506
	60–69	1.1389	1.1587	1.2323	1.2687	0.0407	0.0218	[0.0144, 0.0670]	0.9568
	70–79	0.7265	0.7219	0.7729	0.8021	0.0382	0.0684	[− 0.0072, 0.0836]	0.8679
	80+	0.5543	0.5518	0.5674	0.5872	0.0206	0.0892	[− 0.0078, 0.0490]	0.8296
	**Total**	7.7708	7.8638	8.1199	7.9158	0.0089	0.3955	[− 0.0268, 0.0446]	0.3655
Total		15.2069	15.4817	16.1352	15.9989	0.0199	0.1023	[− 0.0098, 0.0496]	0.8059

This generalized trend is particularly driven by the elder segments (60+) along with young adult females (18–29), since a considerable part of the remaining demographic segments shows signs of slowing down (Wald statistic, Table 3).

A focus on active ingredients reveals that both the volume of dispensed DIDs and the user base of quetiapine, risperidone, olanzapine, aripiprazole and cariprazine have significantly increased, as opposed to the remaining considered drugs, where the trend has remained stable or decreased (Figs. 3 and 8).

Fluctuations in expenditure volume (Fig. 5), encompassing a marked reduction in 2022, align with changes in pricing rather than revisions to state-funded copayment policies (Fig. 6).

### Mood stabilisers

The dispensed DID volume of mood stabilisers show a consistent, albeit moderate, increase, with some demographic sections, predominantly female, showing statistically significant growth rates (Table 4, Fig. 4). Still, the overall increase shows stabilization signs, falling below the 5% significance level.

An individualised analysis of lithium shows a slight but statistically significant consumption increase, which is particularly evident among older males (60 years and older) and in most female groups. When comparing age groups, a steep decline is observed in groups aged 70 and older.

Expenditure volumes (Appendix A) show significant fluctuations, aligned with the lithium price changes.

## Discussion

### Benzodiazepines and zolpidem

Results reveal a sustained high rate of benzodiazepine dispensations, corresponding to broad population-level coverage in Portugal (exceeding 15%) and raising concerns regarding potential misuse^19^. General practicioners (GPs) play a major role in benzodiazepine prescription (Fig. 1), highlighting the importance of training and education policies at the primary care level^20^.

Differences in trends in specific groups may be linked to different stress profiles, changes in lifestyle, or increased recognition and treatment of anxiety disorders in these specific demographic groups^21^. The sharpest, statistically significant year-on-year consumption increase was observed in the 18–29 age group (annual $9.59\%$ rate, $p = 0.0140$ on females and $7.47\%, p = 0.0044$ on males). This may come as a result of various adjustment reactions related to the transition to adult life^22^, suggesting a need for targeted public health interventions.

**Table 4:** Evolution of dispensed DID in mood stabilisers between 2020 and 2022.

Gender	Age group	2020	2021	2022	Mean change per year, fraction of baseline	*p*-value	95% confidence interval	$r^2$
F	18–29	0.1456	0.1554	0.1667	0.0724	0.0254	[0.0356, 0.1092]	0.9984
	30–39	0.2023	0.2035	0.2102	0.0197	0.2450	[− 0.0818, 0.1212]	0.8591
	40–49	0.3778	0.3718	0.3758	− 0.0027	0.7864	[− 0.0996, 0.0942]	0.1084
	50–59	0.4344	0.4525	0.4624	0.0322	0.1086	[− 0.0383, 0.1027]	0.9712
	60–69	0.3577	0.3710	0.3822	0.0342	0.0315	[0.0127, 0.0557]	0.9975
	70–79	0.1860	0.1968	0.2064	0.0551	0.0225	[0.0304, 0.0798]	0.9988
	80+	0.0792	0.0787	0.0787	− 0.0030	0.3778	[− 0.0290, 0.0230]	0.6872
	**Total**	1.7829	1.8297	1.8823	0.0279	0.0218	[0.0158, 0.0400]	0.9988
M	18–29	0.2031	0.2129	0.2103	0.0177	0.4991	[− 0.2060, 0.2414]	0.5014
	30–39	0.2361	0.2365	0.2322	− 0.0083	0.3824	[− 0.0809, 0.0643]	0.6806
	40–49	0.3323	0.3370	0.3337	0.0022	0.8090	[− 0.0871, 0.0915]	0.0873
	50–59	0.3216	0.3348	0.3390	0.0271	0.1859	[− 0.0762, 0.1304]	0.9171
	60–69	0.2352	0.2409	0.2468	0.0248	0.0056	[0.0220, 0.0276]	0.9999
	70–79	0.1373	0.1416	0.1444	0.0258	0.0820	[− 0.0167, 0.0683]	0.9835
	80+	0.0481	0.0491	0.0500	0.0194	0.0075	[0.0165, 0.0223]	0.9999
	**Total**	1.5136	1.5528	1.5564	0.0141	0.2860	[− 0.0723, 0.1005]	0.8114
Total		3.2965	3.3825	3.4387	0.0216	0.0766	[− 0.0115, 0.0547]	0.9856

The consistent pattern of higher benzodiazepine use among women across years, districts and age groups (Fig. 1) seems to reflect differences in healthcare-seeking behaviour and treatment practices, along with various social and psychophysiological factors.

Some districts show higher dispensation in DID than the national average (Fig. 1), including Coimbra, Porto and Viseu, which supports the need for further research into critical correlates and the importance of developing targeted public health interventions. The observed variations across districts could be partially explained by the distribution of psychiatric practice across the country (with well known concentration of mental health professionals in the North of Portugal), yet such covariate is unable to account the lower dispensation rates observed for Lisbon, a region with well-established mental health services.

While diazepam continues to play a role far beyond psychiatry, the widespread use of alprazolam, lorazepam and mexazolam can reflect their established role in the management of panic attacks, insomnia (as a second line of treatment^23^) and generalised anxiety disorder^24,25^. Notwithstanding their role, their long-term use is highly prevalent in Portugal^5^. Risks of long-term benzodiazepines exposure are linked, specially among older adults, to dependence, falls and cognitive impairment. Current guidance recommends short courses only and active deprescribing supported by structured reviews and patient education^26,27^.

The increase in mexazolam dispensation since 2016^7^ appears to reflect changing prescribing practices among healthcare professionals, likely influenced by its long half-life, lower risk of discontinuation effects, and favourable safety profile^28^. Simultaneously, the stabilised number of associated prescriptions further reveals a shift towards prescribing larger quantities/boxes and possibly changes in prescription guidelines affecting how often benzodiazepines can be prescribed.

In the case of zolpidem, although its use has increased slightly, the change was neither consistent nor noticeable across different age and gender groups. The exception is men aged 30–39 years, where, alongside the overall increase in male benzodiazepine use, this pattern may reflect reduced stigma around mental health or point to underlying issues related to stress, anxiety, or sleep disturbances in this age group.

### Antidepressants

The sustained growth in antidepressant dispensations—averaging 7.41% per year since 2019—suggests shifts in mental health treatment patterns in Portugal, reflecting a growing recognition of the importance of addressing depression and anxiety disorders through targeted, treatment-oriented interventions rather than primarily palliative intervention (using benzodiazepines, for example)^29,30^. The observed acceleration since the onset of the pandemic period may be partially attributable to pandemic-related factors, including heightened psychological stress, social isolation, changes in healthcare access, or increased recognition of mental health needs^31^.

While the generalized increase is widespread across all drugs (Fig. 8), age groups and genders (Table 2), it particularly affects young adults (18–29 years), a segment showing a 15% year-on-year increase in dispensed volume (DID) since 2019, highlighting how young adults may be experiencing stressors that drive adjustment responses, such as isolation, economic insecurity and stress. This acceleration, when compared to the pre-pandemic period^7^, is aligned with recent European evidence showing increased antidepressant utilisation among adolescents and young adults during and after the pandemic^11^.

Results further show that antidepressant use is higher among women compared to men (Table 2), which may reflect gender differences in the prevalence of depression^32^, health-seeking behaviours or prescribing practices. Another contributing factor could be the stigma associated with mental illness, which may limit young men’s help-seeking access to psychotherapy and psychiatric care^33^. Results further show higher antidepressant use among older age groups, likely reflecting the mental health impact of medical comorbidities, social isolation, or other age-related stressors^34^. Finally, the data highlight that GPs are the primary prescribers of these medications, underscoring the need for comprehensive training and guidance in primary care settings.

Trazodone, while infrequently prescribed as an antidepressant, shows one of the highest dispensation rates within this class in Portugal (10.9 DID in 2022), highlighting how off-label use can become particularly significant—specifically its widespread use for treating insomnia^35,36^. Sertraline’s effectiveness, safety (including in pregnant women) and extensive approval for anxiety, affective disorders, and trauma- and stressor-related disorders makes the drug very attractive as the first line of treatment by GPs. Escitalopram and fluoxetine are two mature antidepressants that also share a large market. Venlafaxine is the most prescribed serotonin-norepinephrine reuptake inhibitor (SNRI) and seems to be prescribed in more severe depressive episodes with psychomotor inhibition^37^, or as a second line treatment after failed attempts with selective serotonin reuptake inhibitors (SSRIs). The growth of vortioxetine, a recently introduced antidepressant in Portugal (2013^38^), may come as a result of its specific profile in addressing cognitive symptoms in depression, as well as its lack of sexual side effects (which are the second reason—after weight gain—for maladaptation to therapy and dropouts).

Clinically, these trends fit an increase in mental health awareness and education (reducing stigma and improving access to care), as well as a shift towards guideline-concordant care, where antidepressants are combined with psychological therapy for moderate to severe depression, with attention to monitoring, discontinuation symptoms, and relapse prevention^39,40^. At the same time, they may also reflect an increase in adjustment reactions or a lack of early access to counselling and therapy alongside pharmacotherapy. Ongoing research is needed to assess the long-term effects of increased use of antidepressants, including studies of their efficacy, safety and outcomes in different demographic groups.

### Antipsychotics

The generalised increase in antipsychotic use during both the pre-pandemic period and, at a slower rate, pandemic period may suggest a greater recognition and treatment of psychotic disorders and bipolar disorder. However, considering that this trend is more prevalent in middle-aged adult groups (Table 3), we hypothesize that this can be further attributable to non-psychotic and off-label uses, particularly in the management of behavioural and psychological symptoms in different contexts:

- dementia in patients aged 60 and older^41^. Recent large observational studies, however, indicate elevated risks of stroke, venous thromboembolism, myocardial infarction, pneumonia, fractures, and kidney injury in this population, underscoring the importance of regular review and minimization of exposure^42^;
- delirium in the same age groups, despite intensive care unit (ICU) and non-ICU trials showing absent to limited improvement^43,44^;
- adjustment reactions or personality disorders^45^, which may partly explain some of the observed dispensing patterns. including the increase in antipsychotic usage in the female population with age (Fig. 3).

Some of these observations suggest that any observed growth outside psychotic and bipolar indications should be interpreted conservatively.

Quetiapine and olanzapine are among the most widely dispensed antipsychotics, likely due to their broad range of applications. Quetiapine is used in anxiety^46^, off-label for insomnia^47^, and in bipolar disorder^48^, whereas olanzapine is prescribed for bipolar disorder^49^, behavioural control in emergency settings, and delirium^50^. However, their recent stabilisation suggests a possible saturation point or more conservative prescribing, likely due to concerns about side effects. For instance, although some reviews report sleep benefits with quetiapine, most clinical guidelines discourage the use of antipsychotics for primary insomnia because of metabolic and other adverse effects, even at low doses^51–54^, emphasising the need for careful risk-benefit assessment and preference for non-pharmacological alternatives.

The observed recent reduction in expenditure appears to result from price discounts associated with patent expiry, generic entry, and renegotiation for several active substances, including aripiprazole, paliperidone, risperidone, and quetiapine^38^. As long-term medication can impose a substantial financial burden, cost management in health care is essential to ensure accessibility and sustainability of treatments, while enabling investment in innovative therapies.

### Mood stabilisers

The use of mood stabilisers has increased over the period of analysis in some demographic groups, suggesting a greater recognition or diagnosis of conditions requiring mood stabilisation, as well as associated changes in clinical guidelines^55,56^. Additionally, the stress associated with recent global events, such as the COVID-19 pandemic, should not be excluded. It is also possible that increased use in neurology, as a result of changes in epilepsy management guidelines, has played a role in the observed growth^57^.

The reported dispensation metrics of valproic acid and lamotrigine (Fig. 4, Appendix B) must take in consideration their usage as antiepileptics, which may affect the analysis of this class under a psychiatry-centred lens.

Both the volume of total expenditures (Fig. 5) and the state-funded copayment (Fig. 6) were significantly impacted during this period by lithium, as a result of its global price growth registered between 2021 and 2022; with a drop registered in 2023 to prices similar to those in the beginning of 2021. The contribution of lithium dispensation to these expenditure fluctuations is residual, given its consistent and subtle increase.

A segment-focused analysis of lithium dispensation reveals a statistically significant increase among older males and across most female groups. The increase in older men is somewhat paradoxical, given that most guidelines recommend avoiding lithium after age 65 due to its renal complications^58^. However, our results indicate a steep decline in lithium use after age 70, accompanied by an increase in antipsychotic prescriptions, suggesting a risk-benefit reassessment and a consequent substitution with antipsychotics for mood stabilisation in frail patients. The observed increase in women may reflect greater adherence to best practices in mood stabilisation, as alternatives such as valproic acid carry known teratogenic risks^59^. Additionally, the large variation in lithium prices resulting from global market fluctuations could affect localized access to this medication and disrupt management of severe psychiatric conditions. Policymakers and healthcare providers should therefore consider mechanisms to buffer against such price volatility^60–62^.

### Cross-class ancillary trends

Geographically, there is no statistically significant evidence towards a cross-sectional bias in psychiatric drug consumption at the district level (Figs. 1, 2, 3 and 4, district sectioning). However, particular districts, such as Coimbra and Viseu, consistently rank above the national average across consumption metrics for the three most used classes—antidepressants, antipsychotics, and benzodiazepines – while Évora, Beja, and Faro consistently rank below it. At a broader regional level, results indicate higher overall consumption in northern and central districts, particularly for antidepressants and benzodiazepines.

The observed age distributions can be grouped into two main patterns: (i) antidepressants, benzodiazepines, and zolpidem are skewed toward older age groups compared to the national population distribution—more than double in the 80+ group—raising concerns about potential overprescription; in contrast, (ii) antipsychotics and mood stabilisers exhibit similar distributions that peak in the 50–59 age group, with antipsychotics showing an additional, pronounced peak in the 80+ group (Figs. 1, 2, 3 and 4, age sectioning).

The observed high reliance on GPs for prescribing in Portugal (Figs. 1, 2, 3 and 4, medical specialty sectioning) remains consistent with the 2016–2019 period^7^. Antidepressants and benzodiazepines, as well as zolpidem, are predominantly prescribed by these practitioners ($\ge$ 60%), whereas antipsychotics are mainly prescribed by psychiatrists (44%), closely followed by GPs (40%). While lithium is typically prescribed by psychiatrists, other mood stabilisers are largely prescribed by GPs, likely reflecting their use in alternative indications. These patterns underscore the critical importance of ensuring that primary care providers remain well-informed and continuously updated on evidence-based mental health practices.

### Cautionary note

The limited availability of non-pharmacological care in Portugal’s public national health system may channel individuals with moderate distress toward pharmacological treatment and should be considered when interpreting the presented dispensation patterns. We also note limitations inherent in the study design, including the use of dispensation-based data as a proxy for actual consumption, and the location of dispensation acts as a proxy for the users’ geographical location.

## Concluding remarks

Trend and year-on-year analysis of statistical differences confirm a generalised, statistically significant increase in dispensation rates across a broad range of demographic segments since the pandemic onset (Tables 1-4, 6-7). Interrupted time series analysis, comparing 2018–2019 with 2021–2022 to assess pandemic-related effects in level and slope, revealed accelerated antidepressant dispensation growth (Table 8). As increases during this period are likely multifactorial, further research is needed to determine the extent to which they can be attributed to the pandemic context.

These results highlight the need for targeted public health policies, including enhanced psychopharmacological training for general practitioners and strategies to improve coordination with neuropsychiatric practice; the implementation of deprescription practices for the long-term use of specific psychotropic agents—particularly benzodiazepines—in accordance with current guidelines; targeted interventions for the most vulnerable population groups alongside the promotion of psychosocial care, including the integration of psychotherapy services within primary healthcare; and strengthened oversight of the off-label prescription and dispensing of certain psychotropic medications, particularly antipsychotics used in the management of dementia and delirium.

## Supplementary Information

Supplementary Information.

## Figures and Tables

**Fig. 9:**
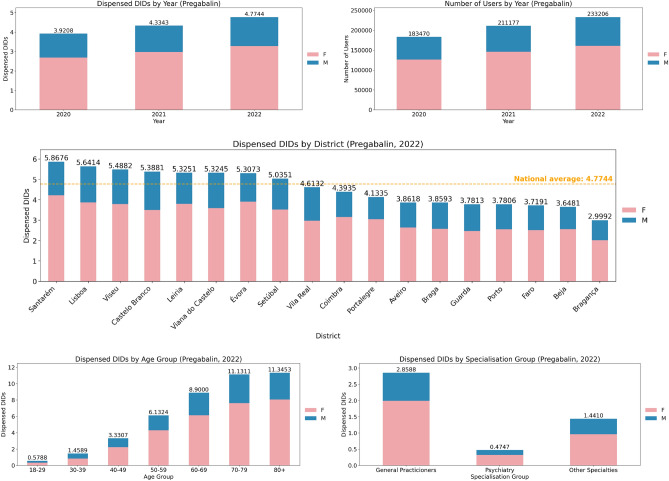
Pregabalin dispensation statistics (DID and user count) across districts, demographic sectors and medical specialties (gender is presented using stacked representations).

## Data Availability

Access to the Portuguese e-prescription and e-dispensation registry must be requested from Serviços Partilhados do Ministério da Saúde (SPMS), accompanied by a clear statement of the study objectives and the relevant ethics approval documentation.

## Appendix C: Pregabalin results and discussion

A statistically significant ($p < 0.05$) increase in the consumption of pregabalin (dispensed DID) has been registered by the majority of demographic segments (predominantly female), a trend followed by the number of active users, as Table 5 and Fig. 9 suggest. The sharp increase in its consumption is most likely driven not only for its anxiolytic properties, but by complementary uses in neurology as an antiepileptic^63^ and in neuropathic pain as an analgesic^64^.

In contrast with other countries, the prescription of pregabalin in Portugal is not subjected to specific regulatory control. Notwithstanding, several recent studies and reviews have highlighted the increasing signals of misuse and dependence associated with gabapentinoids across Europe and beyond, especially among people with comorbid substance use^65–68^. Given the continued rise in pregabalin use, the importance of audits of prescription indications and checks on co-prescribing practices is hereby highlighted. Furthermore, given the critical role of GPs for pregabalin consumption (60% of dispensed DID), we further stress the importance of primary care providers to be well informed and up to date with mental health practices.

**Table 5:** Evolution of dispensed DID in pregabalin between 2019 and 2022.

Gender	Age group	2020	2021	2022	Mean change per year, fraction of baseline	*p*-value	95% confidence interval	$r^2$
F	18–29	0.0311	0.0358	0.0426	0.1860	0.0677	[− 0.0662, 0.4382]	0.9887
	30–39	0.0805	0.0910	0.0966	0.1001	0.1128	[− 0.1276, 0.3278]	0.9689
	40–49	0.2756	0.3040	0.3317	0.1016	0.0040	[0.0934, 0.1098]	1.0000
	50–59	0.5194	0.5736	0.6225	0.0993	0.0189	[0.0619, 0.1367]	0.9991
	60–69	0.6721	0.7417	0.8190	0.1092	0.0191	[0.0677, 0.1507]	0.9991
	70–79	0.6487	0.7256	0.8103	0.1245	0.0180	[0.0797, 0.1693]	0.9992
	80+	0.4656	0.5079	0.5603	0.1018	0.0390	[0.0224, 0.1812]	0.9962
	Total	2.6931	2.9796	3.2830	0.1095	0.0105	[0.0866, 0.1324]	0.9997
M	18–29	0.0228	0.0239	0.0303	0.1653	0.2438	[− 0.6808, 1.0114]	0.8603
	30–39	0.0576	0.0631	0.0695	0.1035	0.0236	[0.0548, 0.1522]	0.9986
	40–49	0.1335	0.1504	0.1593	0.0968	0.1123	[− 0.1225, 0.3161]	0.9692
	50–59	0.2140	0.2391	0.2654	0.1200	0.0090	[0.0984, 0.1416]	0.9998
	60–69	0.3031	0.3366	0.3665	0.1046	0.0207	[0.0614, 0.1478]	0.9989
	70–79	0.3088	0.3349	0.3723	0.1027	0.0657	[− 0.0325, 0.2379]	0.9894
	80+	0.1879	0.2067	0.2281	0.1070	0.0238	[0.0562, 0.1578]	0.9986
	Total	1.2277	1.3547	1.4914	0.1074	0.0135	[0.0783, 0.1365]	0.9995
Total		3.9208	4.3343	4.7744	0.1089	0.0114	[0.0840, 0.1338]	0.9997

## Appendix D: Time-aware statistics

**Table 6:** Trend analysis of dispensation DID per psychotropic group and demography: (i) comparison of annual slopes of pre-pandemic and pandemic periods: $\frac{DID 2019}{DID 2018}$ *vs* $\frac{DID 2022}{DID 2021}$ and $\frac{DID 2019}{DID 2017}$ *vs* $\frac{DID 2022}{DID 2020}$; (ii) *p*-values from Wald testing ($H_0: slope = 0$) from 2016 to 2022.

	*Slope ratios*												*Wald test* (*p*-value)		
	Antidepressant		Antipsychotic		Benzodiazepine		Antidepressant		Antipsychotic		Benzodiazepine		Antidep.	Antipsyc.	Benzod.
	2018–19	2021–22	2018–19	2021–22	2018–19	2021–22	2017–19	2020–22	2017–19	2020–22	2017–19	2020–22	2016–22	2016–22	2016–22
18–29	1.1237	1.1845	1.1188	0.9771	1.0621	1.0439	1.2168	1.5085	1.1875	1.0179	1.0573	1.1401	<0.001	0.003	<0.001
30–39	1.0314	1.0872	1.0820	0.9318	1.0194	0.9747	1.0345	1.2049	1.0672	0.9292	0.9752	0.9730	<0.001	0.088	0.047
40–49	1.0601	1.0597	1.0851	0.9475	1.0551	0.9760	1.1068	1.1605	1.0985	0.9556	1.0409	0.9926	<0.001	0.032	0.004
50–59	1.0613	1.0715	1.0930	0.9818	1.0622	0.9918	1.1095	1.1666	1.1304	1.0322	1.0588	1.0191	<0.001	0.003	0.001
60–69	1.0691	1.0664	1.0907	1.0298	1.0707	1.0017	1.1359	1.1564	1.1524	1.0980	1.0701	1.0338	<0.001	<0.001	<0.001
70–79	1.0898	1.0841	1.0860	1.0371	1.0845	1.0069	1.1773	1.1844	1.1621	1.1155	1.0931	1.0475	<0.001	<0.001	<0.001
80+	1.1081	1.0734	1.0934	1.0318	1.1068	0.9912	1.2336	1.1619	1.1929	1.0856	1.1342	1.0116	<0.001	0.003	0.004
F	1.0722	1.0743	1.0801	1.0084	1.0744	0.9926	1.1370	1.1770	1.1323	1.0610	1.0706	1.0210	<0.001	0.002	0.001
M	1.0870	1.0864	1.1014	0.9749	1.0721	1.0024	1.1691	1.1932	1.1391	1.0066	1.0851	1.0314	<0.001	0.007	0.001
Total	1.0758	1.0773	1.0909	0.9915	1.0737	0.9956	1.1446	1.1809	1.1357	1.0334	1.0750	1.0242	<0.001	0.004	0.001

**Table 7:** Statistical testing of changes in dispensation DID across periods using Wilcoxon signed-rank on estimates obtained from paired age groups (one-tailed testing for statistically significant increases). Two scenarios: (i) averaged DID from pre-pandemic and pandemic periods, and (ii) yearly changes along the pandemic period.

*Paired testing* (*p*-values)	2018–19 vs 2021–22	2020 vs 2019	2021 vs 2020	2022 vs 2021
Antidepressant	<0.01	<0.01	<0.01	<0.01
Antipsychotic	$<$ $<$0.01	<0.01	<0.01	0.656
Benzodiazepine	<0.01	<0.01	<0.01	0.851
Mood stabiliser	–	-	0.023	<0.01
Pregabalin	-	-	<0.01	<0.01

**Table 8:** Interrupted time series analysis of increases before (2018–2019) and after the pandemic (2021–2022) where 2020 is defined as the intervention time via OLS regression using estimates obtained from paired age groups. The assessed coefficients: *intercept* ($\beta _0$) is the estimated level of the outcome at the start of the series; *time* ($\beta _1$) is the pre-intervention trend, *intervention* ($\beta _2$) is the immediate level change between 2019 and 2021, and *time after intervention* ($\beta _3$) is the rate of change of the slope against the pre-pandemic period.

Interrupted time series (coefficients)	Antidepressants	Antipsychotics	Benzodiazepines
Constant (intercept)	13.8018	1.8103	8.8605
Time	1.1314	0.1810	0.7052
Intervention	0.6379	0.1521	0.7093
Time after intervention	0.2676	− 0.2005	− 0.7529

## Appendix E: List of drugs and medical specialties

### List of drugs

- *Benzodiazepines and similar drugs*: alprazolam, bromazepam, brotizolam, cetazolam, clobazam, clorazepate dipotassium, chlordiazepoxide (without or with clidinium bromide), cloxazolam, diazepam, estazolam, flurazepam, loprazolam, lorazepam, mexazolam, midazolam, oxazepam, prazepam, temazepam, triazolam, zolpidem.
- *Antidepressants*:
  - $\alpha$− 2 *blockers*: mianserine, mirtazapine, trazodone;
  - *Selective serotonin reuptake inhibitors* (SSRIs): citalopram, escitalopram, fluoxetine, fluxovamine, paroxetine, sertraline, vortioxetine;
  - *Serotonin-norepinephrine reuptake inhibitors* (SNRIs): duloxetine, milnacipran, venlafaxine, reboxetine;
  - *Monoamine oxidase inhibitor* (MAOI): moclobemide;
  - *Tricyclic antidepressants* (TCAs): amitriptiline, clomipramine, dosulepine, imipramine, maprotiline, nortriptyline, pirlindole, trimipramine.
- *Antipsychotics*:
  - *First-generation* (typical): ciamemazine, chlorpromazine, flupentixol, haloperidol, levomepromazine, pimozide;
  - *Second-generation* (atypical): amisulpride, aripiprazole, cariprazine*, clozapine*, melperone, olanzapine, paliperidone, quetiapine, risperidone, sulpiride, tiapride, ziprasidone, zotepine, zuclopenthixol.
- *Mood stabilisers*: Lamotrigine*, lithium*, valproic acid*.

*****drug only present in dispensation data starting in 2020.

### List of medical specialties

- *Psychiatry*
- *General practice*
- *Others:* Anatomical pathology, anaesthesiology, angiology and vascular surgery, cardiology, paediatric cardiology, cardiothoracic surgery, general surgery, maxillofacial surgery, paediatric surgery, dermatology and venereology, endocrinology and nutrition, stomatology, gastroenterology, medical genetics, obstetrics and gynaecology, clinical haematology, immunology and allergology, immunohaematology, infectious diseases, dentistry, sports medicine, occupational medicine, physiatry, general internal medicine, legal medicine, nuclear medicine, tropical medicine, nephrology, neurosurgery, neurology, neuroradiology, ophthalmology, oncology, orthopaedics, otorhinolaryngology, clinical pathology, paediatrics, plastic, reconstructive and aesthetic surgery, pulmonology, paediatric psychiatry, radiology, radiation oncology, rheumatology, public health, physical service, urology

## References

[1] Eurofound. Share of adults at risk of depression in 2020–22, Living, working and COVID-19 e-survey. https://stat.link/qvcu7m.

[2] The Lancet Psychiatry. Global burden of disease 2021: mental health messages. *The Lancet Psychiatry* **11**, 573. 10.1016/S2215-0366(24)00222-0 (2024). 39025623 10.1016/S2215-0366(24)00222-0

[3] Institute of Health Metrics and Evaluation. Global Health Data Exchange. https://vizhub.healthdata.org/gbd-results/ (2019) .

[4] OECD. *Consumption of medicines for selected chronic conditions, 2011, 2019 and 2021 (or nearest years)*. https://www.oecd-ilibrary.org/content/component/f11c6179-en (2023).

[5] Gomes, S. et al. Prescrição de benzodiazepinas e outros sedativos na administração regional de saúde de lisboa e vale do tejo de 2013 a 2020: Um estudo retrospetivo. *Acta Med. Port.* **36**, 264–274 (2023). 37029641 10.20344/amp.18680

[6] OECD & Observatory, E. *Health at a Glance 2023 *(2023).

[7] Madeira, L., Queiroz, G. & Henriques, R. Prepandemic psychotropic drug status in Portugal: a nationwide pharmacoepidemiological profile. *Sci. Rep.* **13**, 6912 (2023). 37106018 10.1038/s41598-023-33765-0 PMC10139661

[8] Vukićević, T., Draganić, P., Škribulja, M., Puljak, L. & Došenović, S. Consumption of psychotropic drugs in Croatia before and during the COVID-19 pandemic: a 10-year longitudinal study (2012–2021). *Soc. Psychiatry Psychiatric Epidemiol.* 1–13 (2023). 10.1007/s00127-023-02574-1 37847256

[9] Benistand, P. et al. Effect of the COVID-19 pandemic on the psychotropic drug consumption. *Front. Psych.* **13**, 1020023 (2022). 10.3389/fpsyt.2022.1020023 PMC9797694 36590615

[10] Laurin, A. et al. Psychotropic drugs consumption during 2020 COVID-19 pandemic and lockdowns: evidence of a surprising resilience of the drugs delivery system in France. *Eur. Neuropsychopharmacol.* **73**, 48–61 (2023). 37119562 10.1016/j.euroneuro.2023.04.004 PMC10086109

[11] García, M. L. N., Martínez, P. F., Bretón, E. F., Martínez Alfonso, M. M. & Gil, P. S. Psychotropic consumption before and during COVID-19 in Asturias, Spain. *BMC Public Health* **23**, 494 (2023). 36918825 10.1186/s12889-023-15360-0 PMC10014411

[12] Del Fiol, F. d. S., Bergamaschi, C. d. C. & Barberato-Filho, S. Sales trends of psychotropic drugs in the COVID-19 pandemic: a national database study in Brazil. *Front. Pharmacol.* **14**, 1131357 (2023). 37007033 10.3389/fphar.2023.1131357 PMC10063839

[13] Oscoz-Irurozqui, M., Villani, L., Martinelli, S., Ricciardi, W. & Gualano, M. R. Trend analysis of antidepressant consumption in Italy from 2008 to 2022 in a public health perspective. *Sci. Rep.* **15**, 12124 (2025). 40204785 10.1038/s41598-025-96037-z PMC11982533

[14] Newman, H., Branford, D., Laugharne, R., Byng, R. & Shankar, R. Twenty-five year trend in antipsychotic medication prescribing in England: challenges and opportunities. *BJPsych Open* **11**, e151 (2025). 40665640 10.1192/bjo.2025.10073 PMC12303834

[15] Carrasco-Garrido, P. et al. Time trend in psychotropic medication use in Spain: a nationwide population-based study. *Int. J. Environ. Res. Public Health* **13**, 1177 (2016). 27886138 10.3390/ijerph13121177 PMC5201318

[16] De Wilde, J., Geerts, S., Van Dorpe, J., Bruhwyler, J. & Géczy, J. A double-blind randomized placebo-controlled study of the efficacy and safety of pirlindole, a reversible monoamine oxidase a inhibitor, in the treatment of depression. *Acta Psychiatr. Scand.* **94**, 404–410 (1996). 9020990 10.1111/j.1600-0447.1996.tb09881.x

[17] Macedo, A., Leiria, E. & Filipe, A. Pirlindole in the treatment of depression: a meta-analysis. *Clin. Drug Investig.* **31**, 61–71 (2011). 21053988 10.2165/11586690-000000000-00000

[18] Instituto Nacional de Estatística. Instituto Nacional de Estatística. https://www.ine.pt/.

[19] Sarangi, A., McMahon, T. & Gude, J. Benzodiazepine misuse: an epidemic within a pandemic. *Cureus* **13** (2021). 10.7759/cureus.15816 PMC8294026 34306882

[20] Seeman, M. V. Men and women respond differently to antipsychotic drugs. *Neuropharmacology* **163**, 107631 (2020). 31077728 10.1016/j.neuropharm.2019.05.008

[21] Lukačišinová, A. et al. The prevalence and prescribing patterns of benzodiazepines and Z-drugs in older nursing home residents in different European countries and Israel: retrospective results from the EU SHELTER study. *BMC Geriatr.* **21**, 1–16 (2021). 33902474 10.1186/s12877-021-02213-x PMC8077828

[22] HSBC - Health Behaviour in School-aged Children. A saúde dos adolescentes portugueses em contexto de pandemia – dados nacionais 2022. https://hbsc.org/portuguese-adolescents-mental-health-and-well-being-decline-hbsc-study-reveals.

[23] Riemann, D. et al. The European insomnia guideline: an update on the diagnosis and treatment of insomnia 2023. *J. Sleep Res.* **32**, e14035 (2023). 38016484 10.1111/jsr.14035

[24] Ait-Daoud, N., Hamby, A. S., Sharma, S. & Blevins, D. A review of alprazolam use, misuse, and withdrawal. *J. Addict. Med.* **12**, 4–10 (2018). 28777203 10.1097/ADM.0000000000000350 PMC5846112

[25] Soyka, M. et al. Long-term use of benzodiazepines in chronic insomnia: a European perspective. *Front. Psych.* **14**, 1212028 (2023). 10.3389/fpsyt.2023.1212028 PMC10433200 37599882

[26] Brandt, J. et al. Prescribing and deprescribing guidance for benzodiazepine and benzodiazepine receptor agonist use in adults with depression, anxiety, and insomnia: an international scoping review. *EClinicalMedicine* **70** (2024). 10.1016/j.eclinm.2024.102507 PMC10955669 38516102

[27] Shapoval, V. et al. Barriers to deprescribing benzodiazepines in older adults in a survey of European physicians. *JAMA Netw. Open* **8**, e2459883–e2459883 (2025). 40029661 10.1001/jamanetworkopen.2024.59883 PMC11877185

[28] Fernandes, H. & Moreira, R. Mexazolam: clinical efficacy and tolerability in the treatment of anxiety. *Neurol. Therapy* **3**, 1–14 (2014). 10.1007/s40120-014-0016-7 PMC4381915 26000220

[29] Dell’Osso, B., Buoli, M., Baldwin, D. S. & Altamura, A. C. Serotonin norepinephrine reuptake inhibitors (SNRIs) in anxiety disorders: a comprehensive review of their clinical efficacy. *Hum. Psychopharmacol. Clin. Exp.* **25**, 17–29 (2010). 10.1002/hup.1074 20041476

[30] Jakubovski, E., Johnson, J. A., Nasir, M., Müller-Vahl, K. & Bloch, M. H. Systematic review and meta-analysis: Dose-response curve of SSRIs and SNRIs in anxiety disorders. *Depress. Anxiety* **36**, 198–212 (2019). 30479005 10.1002/da.22854

[31] De Bandt, D., Haile, S. R., Devillers, L., Bourrion, B. & Menges, D. Prescriptions of antidepressants and anxiolytics in France 2012–2022 and changes with the COVID-19 pandemic: interrupted time series analysis. *BMJ Mental Health* **27** (2024). 10.1136/bmjment-2024-301026 PMC10900346 38413052

[32] Hyde, J. S. & Mezulis, A. H. Gender differences in depression: biological, affective, cognitive, and sociocultural factors. *Harv. Rev. Psychiatry* **28**, 4–13 (2020). 31913978 10.1097/HRP.0000000000000230

[33] Lynch, L., Long, M. & Moorhead, A. Young men, help-seeking, and mental health services: exploring barriers and solutions. *Am. J. Mens Health* **12**, 138–149 (2018). 27365212 10.1177/1557988315619469 PMC5734535

[34] Batelaan, N., Scholten, W., Rhebergen, D. & Van Balkom, A. Why we need to evaluate long-term antidepressant use in older patients with depression. *Age Ageing* **50**, 690–692 (2021). 33951160 10.1093/ageing/afaa286

[35] Nierenberg, A. A., Adler, L. A., Peselow, E., Zornberg, G. & Rosenthal, M. Trazodone for antidepressant-associated insomnia. *Am. J. Psychiatry* **151**, 1069–1072 (1994). 8010365 10.1176/ajp.151.7.1069

[36] Jaffer, K. Y. et al. Trazodone for insomnia: a systematic review. *Innov. Clin. Neurosci.* **14**, 24 (2017). 29552421 PMC5842888

[37] Chokkakula, S. et al. The post-marketing safety of venlafaxine: a real-world two-decade pharmacovigilance study using the FAERS database. *Front. Pharmacol.* **17**, 1737113 (2026). 41646188 10.3389/fphar.2026.1737113 PMC12868114

[38] INFARMED - Autoridade Nacional do Medicamento e Produtos de Saúde. Sistema de Preços de Referência. https://www.infarmed.pt/web/infarmed/entidades/medicamentos-uso-humano/avaliacao-tecnologias-saude/avaliacao-terapeutica-e-economica/sistema-de-precos-de-referencia.

[39] National Institute for Health and Care Excellence (NICE). *Depression in adults: treatment and management (NG222)*. London, UK. https://www.nice.org.uk/guidance/ng222. Published 29 June 2022; last reviewed 19 September 2024 (2022). 35977056

[40] Mosiołek, A. & Podlecka, M. Pharmacotherapy and psychotherapy in depression-complementarity or exclusion?. *Adv. Psychiatry Neurol./Postępy Psychiatrii i Neurologii* **33**, 257–266 (2024). 10.5114/ppn.2024.147104 PMC11891753 40070429

[41] Wetzels, R., Zuidema, S. U., de Jonghe, J. F., Verhey, F. R. & Koopmans, R. Prescribing pattern of psychotropic drugs in nursing home residents with dementia. *Int. Psychogeriatr.* **23**, 1249–1259 (2011). 21682938 10.1017/S1041610211000755

[42] Mok, P. L. et al. Multiple adverse outcomes associated with antipsychotic use in people with dementia: population based matched cohort study. *BMJ* **385** (2024). 10.1136/bmj-2023-076268 PMC11022137 38631737

[43] Carayannopoulos, K. L. et al. Antipsychotics in the treatment of delirium in critically ill patients: a systematic review and meta-analysis of randomized controlled trials. *Crit. Care Med.* **52**, 1087–1096 (2024). 38488422 10.1097/CCM.0000000000006251

[44] Burry, L. et al. Antipsychotics for treatment of delirium in hospitalised non-ICU patients. *Cochrane Database Syst. Rev.* (2018). 10.1002/14651858.CD005594.pub3 PMC6513380 29920656

[45] Maglione, M. et al. Off-label use of atypical antipsychotics: an update (2011). 22132426

[46] Maneeton, N. et al. Quetiapine monotherapy in acute treatment of generalized anxiety disorder: a systematic review and meta-analysis of randomized controlled trials. *Drug Design, Dev. Therapy* 259–276 (2016). 10.2147/DDDT.S89485 PMC4716733 26834458

[47] Karsten, J., Hagenauw, L. A., Kamphuis, J. & Lancel, M. Low doses of mirtazapine or quetiapine for transient insomnia: a randomised, double-blind, cross-over, placebo-controlled trial. *J. Psychopharmacol.* **31**, 327–337 (2017). 28093029 10.1177/0269881116681399

[48] Ketter, T. A., Miller, S., Dell’Osso, B. & Wang, P. W. Treatment of bipolar disorder: review of evidence regarding quetiapine and lithium. *J. Affect. Disord.* **191**, 256–273 (2016). 26688495 10.1016/j.jad.2015.11.002

[49] Cipriani, A., Rendell, J. & Geddes, J. R. Olanzapine in the long-term treatment of bipolar disorder: a systematic review and meta-analysis. *J. Psychopharmacol.* **24**, 1729–1738 (2010). 19828571 10.1177/0269881109106900

[50] Skrobik, Y. K., Bergeron, N., Dumont, M. & Gottfried, S. B. Olanzapine vs haloperidol: treating delirium in a critical care setting. *Intensive Care Med.* **30**, 444–449 (2004). 14685663 10.1007/s00134-003-2117-0

[51] Lin, C.-Y., Chiang, C.-H., Tseng, M.-C.M., Tam, K.-W. & Loh, E.-W. Effects of quetiapine on sleep: a systematic review and meta-analysis of clinical trials. *Eur. Neuropsychopharmacol.* **67**, 22–36 (2023). 36463762 10.1016/j.euroneuro.2022.11.008

[52] Fauska, C. et al. Effects of the antipsychotic quetiapine on sleep and breathing: a review of clinical findings and potential mechanisms. *J. Sleep Res.* **33**, e14051 (2024). 37833613 10.1111/jsr.14051

[53] Modesto-Lowe, V., Harabasz, A. K. & Walker, S. A. Quetiapine for primary insomnia: consider the risks. *Clevel. Clin. J. Med.* **88**, 286–294 (2021). 10.3949/ccjm.88a.20031 33941603

[54] Cinar, B. G. et al. Patterns and indications for quetiapine prescribing in Dutch primary care: a retrospective database study. *BJGP Open* **9** (2025). 10.3399/BJGPO.2024.0219 PMC12728850 40730475

[55] O’Donovan, C. & Alda, M. Depression preceding diagnosis of bipolar disorder. *Front. Psych.* **11**, 537846 (2020). 10.3389/fpsyt.2020.00500 PMC7300293 32595530

[56] Lucidi, L. et al. Gut microbiota and bipolar disorder: an overview on a novel biomarker for diagnosis and treatment. *Int. J. Mol. Sci.* **22**, 3723 (2021). 33918462 10.3390/ijms22073723 PMC8038247

[57] Kanner, A. M. et al. Mood disorders in adults with epilepsy: a review of unrecognized facts and common misconceptions. *Ann. Gen. Psychiatry* **23**, 11 (2024). 38433207 10.1186/s12991-024-00493-2 PMC10910742

[58] Schoretsanitis, G. et al. Prevalence of impaired kidney function in patients with long-term lithium treatment: a systematic review and meta-analysis. *Bipolar Disord.* **24**, 264–274 (2022). 34783413 10.1111/bdi.13154

[59] Steinbart, D., Gaus, V., Kowski, A. B. & Holtkamp, M. Valproic acid use in fertile women with genetic generalized epilepsies. *Acta Neurol. Scand.* **144**, 288–295 (2021). 33977526 10.1111/ane.13446

[60] U.S. Geological Survey. Mineral commodity summaries 2024. Tech. Rep. 2024, U.S. Geological Survey, Reston, VA, USA. https://pubs.usgs.gov/periodicals/mcs2024/mcs2024.pdf (2024).

[61] U.S. Geological Survey. Mineral commodity summaries 2025. Tech. Rep. 2025, U.S. Geological Survey, Reston, VA, USA. https://pubs.usgs.gov/periodicals/mcs2025/mcs2025.pdf (2025).

[62] International Energy Agency (IEA). Global critical minerals outlook 2024. https://www.iea.org/reports/global-critical-minerals-outlook-2024. Licence: CC BY 4.0 (2024).

[63] Hamandi, K. & Sander, J. W. Pregabalin: a new antiepileptic drug for refractory epilepsy. *Seizure* **15**, 73–78 (2006). 16413993 10.1016/j.seizure.2005.11.005

[64] Blommel, M. L. & Blommel, A. L. Pregabalin: an antiepileptic agent useful for neuropathic pain. *Am. J. Health Syst. Pharm.* **64**, 1475–1482 (2007). 17617497 10.2146/ajhp060371

[65] McNeilage, A. G., Sim, A., Nielsen, S., Murnion, B. & Ashton-James, C. E. Experiences of misuse and symptoms of dependence among people who use gabapentinoids: a qualitative systematic review. *Int. J. Drug Policy* **133**, 104605 (2024). 39388918 10.1016/j.drugpo.2024.104605

[66] Soeiro, T., Uras, M., Jouanjus, É., Lapeyre-Mestre, M. & Micallef, J. High-dose use of pregabalin and Gabapentin in France: a retrospective, population-based cohort study. *The Lancet Reg. Health–Eur.* **57** (2025). 10.1016/j.lanepe.2025.101424 PMC12355116 40823189

[67] Ghosh, A. et al. Understanding pregabalin misuse and dependence: Insights from a north Indian addiction treatment center. *Indian J. Psych.* **66**, 723–728 (2024). 10.4103/indianjpsychiatry.indianjpsychiatry_307_24 PMC11469571 39398509

[68] Luo, Y. et al. A case report of pregabalin misuse leading to drug dependence. *Front. Psych.* **16**, 1511168 (2025). 10.3389/fpsyt.2025.1511168 PMC11969338 40191116

