# Supplementary material for: Patterns of psychotropic drug dispensation in Portugal amidst the COVID-19 pandemic and beyond
Source: Sci Rep. 2026 May 20;16:22892. doi: 10.1038/s41598-026-50987-0 (PMC13388679; doi:10.1038/s41598-026-50987-0)
Supplement: Supplementary file 1 — Supplementary Information. [file 41598_2026_50987_MOESM1_ESM.pdf]

SUPPLEMENT: LIST OF AVAILABLE DRUG PACKAGES

Table S1. Drug packages by class, active ingredient, administration route, packaging details, and dosage (including DDDs per package).

Drug class	Drug name	ATC code	DDD	Mode	Package type	Dosage/unit	Total dosage (g)	DDD/package
**Antidepressant**	Amitriptyline	N06AA09	75 mg	Coated tablet	Blister 10 unit(s)	10 mg	0.1000	1.333
	Amitriptyline	N06AA09	75 mg	Coated tablet	Blister 20 unit(s)	10 mg	0.2000	2.667
	Amitriptyline	N06AA09	75 mg	Coated tablet	Blister 60 unit(s)	10 mg	0.6000	8.000
	Amitriptyline	N06AA09	75 mg	Coated tablet	Blister 60 unit(s)	25 mg	1.5000	20.000
	Amitriptyline	N06AA09	75 mg	Coated tablet	Blister 60 unit(s)	75 mg	4.5000	60.000
	Citalopram	N06AB04	20 mg	Film-coated tablet	Blister 10 unit(s)	10 mg	0.1000	5.000
	Citalopram	N06AB04	20 mg	Film-coated tablet	Blister 14 unit(s)	10 mg	0.1400	7.000
	Citalopram	N06AB04	20 mg	Film-coated tablet	Blister 14 unit(s)	20 mg	0.2800	14.000
	Citalopram	N06AB04	20 mg	Film-coated tablet	Blister 28 unit(s)	40 mg	1.1200	56.000
	Citalopram	N06AB04	20 mg	Film-coated tablet	Blister 30 unit(s)	40 mg	1.2000	60.000
	Citalopram	N06AB04	20 mg	Film-coated tablet	Blister 56 unit(s)	10 mg	0.5600	28.000
	Citalopram	N06AB04	20 mg	Film-coated tablet	Blister 56 unit(s)	20 mg	1.1200	56.000
	Citalopram	N06AB04	20 mg	Film-coated tablet	Blister 60 unit(s)	10 mg	0.6000	30.000
	Citalopram	N06AB04	20 mg	Film-coated tablet	Blister 60 unit(s)	20 mg	1.2000	60.000
	Clomipramine	N06AA04	100 mg	Extended-release tablet	Blister 60 unit(s)	75 mg	4.5000	45.000
	Clomipramine	N06AA04	100 mg	Coated tablet	Blister 10 unit(s)	10 mg	0.1000	1.000
	Clomipramine	N06AA04	100 mg	Coated tablet	Blister 60 unit(s)	10 mg	0.6000	6.000
	Clomipramine	N06AA04	100 mg	Coated tablet	Blister 60 unit(s)	25 mg	1.5000	15.000
	Clomipramine	N06AA04	100 mg	Injectable solution	Ampoule 5 unit(s) 2 ml	25 mg/2 ml	0.1250	1.250
	Dosulepin	N06AA16	150 mg	Coated tablet	Blister 20 unit(s)	75 mg	1.5000	10.000
	Dosulepin	N06AA16	150 mg	Coated tablet	Blister 60 unit(s)	75 mg	4.5000	30.000
	Duloxetine	N06AX21	60 mg	Gastro-resistant capsule	Blister 28 unit(s)	120 mg	3.3600	56.000
	Duloxetine	N06AX21	60 mg	Gastro-resistant capsule	Blister 28 unit(s)	30 mg	0.8400	14.000
	Duloxetine	N06AX21	60 mg	Gastro-resistant capsule	Blister 28 unit(s)	60 mg	1.6800	28.000
	Duloxetine	N06AX21	60 mg	Gastro-resistant capsule	Blister 28 unit(s)	90 mg	2.5200	42.000
	Duloxetine	N06AX21	60 mg	Gastro-resistant capsule	Blister 7 unit(s)	30 mg	0.2100	3.500
	Escitalopram	N06AB10	10 mg	Orodispersible tablet	Blister 56 unit(s)	10 mg	0.5600	56.000
	Escitalopram	N06AB10	10 mg	Orodispersible tablet	Blister 56 unit(s)	20 mg	1.1200	112.000
	Escitalopram	N06AB10	10 mg	Film-coated tablet	Blister 14 unit(s)	10 mg	0.1400	14.000
	Escitalopram	N06AB10	10 mg	Film-coated tablet	Blister 20 unit(s)	10 mg	0.2000	20.000
	Escitalopram	N06AB10	10 mg	Film-coated tablet	Blister 56 unit(s)	10 mg	0.5600	56.000
	Escitalopram	N06AB10	10 mg	Film-coated tablet	Blister 56 unit(s)	20 mg	1.1200	112.000
	Escitalopram	N06AB10	10 mg	Film-coated tablet	Blister 60 unit(s)	10 mg	0.6000	60.000
	Escitalopram	N06AB10	10 mg	Film-coated tablet	Blister 60 unit(s)	20 mg	1.2000	120.000
	Escitalopram	N06AB10	10 mg	Oral drops	Dropper bottle 1x 15 ml	20 mg/ml	0.3000	30.000
	Fluoxetine	N06AB03	20 mg	Capsule	Blister 10 unit(s)	20 mg	0.2000	10.000
	Fluoxetine	N06AB03	20 mg	Capsule	Blister 14 unit(s)	20 mg	0.2800	14.000
	Fluoxetine	N06AB03	20 mg	Capsule	Blister 20 unit(s)	20 mg	0.4000	20.000
	Fluoxetine	N06AB03	20 mg	Capsule	Blister 28 unit(s)	20 mg	0.5600	28.000
	Fluoxetine	N06AB03	20 mg	Capsule	Blister 50 unit(s)	20 mg	1.0000	50.000
	Fluoxetine	N06AB03	20 mg	Capsule	Blister 56 unit(s)	20 mg	1.1200	56.000
	Fluoxetine	N06AB03	20 mg	Capsule	Blister 60 unit(s)	20 mg	1.2000	60.000
	Fluoxetine	N06AB03	20 mg	Oral solution	Bottle 1x 140 ml	4 mg/ml	0.5600	28.000
	Fluvoxamine	N06AB08	100 mg	Film-coated tablet	Blister 10 unit(s)	50 mg	0.5000	5.000
	Fluvoxamine	N06AB08	100 mg	Film-coated tablet	Blister 20 unit(s)	50 mg	1.0000	10.000
	Fluvoxamine	N06AB08	100 mg	Film-coated tablet	Blister 60 unit(s)	100 mg	6.0000	60.000
	Fluvoxamine	N06AB08	100 mg	Film-coated tablet	Blister 60 unit(s)	50 mg	3.0000	30.000
	Imipramine	N06AA02	100 mg	Coated tablet	Blister 60 unit(s)	10 mg	0.6000	6.000
	Imipramine	N06AA02	100 mg	Coated tablet	Blister 60 unit(s)	25 mg	1.5000	15.000
	Maprotiline	N06AA21	100 mg	Coated tablet	Blister 10 unit(s)	25 mg	0.2500	2.500
	Maprotiline	N06AA21	100 mg	Coated tablet	Blister 60 unit(s)	25 mg	1.5000	15.000
	Maprotiline	N06AA21	100 mg	Coated tablet	Blister 60 unit(s)	50 mg	3.0000	30.000
	Maprotiline	N06AA21	100 mg	Coated tablet	Blister 60 unit(s)	75 mg	4.5000	45.000
	Maprotiline	N06AA21	100 mg	Film-coated tablet	Blister 60 unit(s)	25 mg	1.5000	15.000
	Maprotiline	N06AA21	100 mg	Film-coated tablet	Blister 60 unit(s)	50 mg	3.0000	30.000
	Maprotiline	N06AA21	100 mg	Film-coated tablet	Blister 60 unit(s)	75 mg	4.5000	45.000
	Mianserine	N06AX03	60 mg	Coated tablet	Blister 10 unit(s)	30 mg	0.3000	5.000
	Mianserine	N06AX03	60 mg	Coated tablet	Blister 60 unit(s)	30 mg	1.8000	30.000
	Milnacipran	N06AX17	100 mg	Capsule	Blister 56 unit(s)	25 mg	1.4000	14.000
	Milnacipran	N06AX17	100 mg	Capsule	Blister 56 unit(s)	50 mg	2.8000	28.000
	Mirtazapine	N06AX11	30 mg	Orodispersible tablet	Blister 18 unit(s)	15 mg	0.2700	9.000
	Mirtazapine	N06AX11	30 mg	Orodispersible tablet	Blister 20 unit(s)	15 mg	0.3000	10.000
	Mirtazapine	N06AX11	30 mg	Orodispersible tablet	Blister 20 unit(s)	30 mg	0.6000	20.000
	Mirtazapine	N06AX11	30 mg	Orodispersible tablet	Blister 30 unit(s)	15 mg	0.4500	15.000
	Mirtazapine	N06AX11	30 mg	Orodispersible tablet	Blister 30 unit(s)	30 mg	0.9000	30.000
	Mirtazapine	N06AX11	30 mg	Orodispersible tablet	Blister 30 unit(s)	45 mg	1.3500	45.000
	Mirtazapine	N06AX11	30 mg	Orodispersible tablet	Blister 60 unit(s)	15 mg	0.9000	30.000
	Mirtazapine	N06AX11	30 mg	Orodispersible tablet	Blister 60 unit(s)	30 mg	1.8000	60.000
	Mirtazapine	N06AX11	30 mg	Orodispersible tablet	Blister 6 unit(s)	15 mg	0.0900	3.000
	Mirtazapine	N06AX11	30 mg	Orodispersible tablet	Thermowelded tape 30 units	15 mg	0.4500	15.000
	Mirtazapine	N06AX11	30 mg	Orodispersible tablet	Thermowelded tape 30 units	30 mg	0.9000	30.000
	Mirtazapine	N06AX11	30 mg	Orodispersible tablet	Thermowelded tape 6 units	15 mg	0.0900	3.000
	Mirtazapine	N06AX11	30 mg	Coated tablet	Blister 10 unit(s)	30 mg	0.3000	10.000
	Mirtazapine	N06AX11	30 mg	Coated tablet	Blister 14 unit(s)	15 mg	0.2100	7.000
	Mirtazapine	N06AX11	30 mg	Coated tablet	Blister 30 unit(s)	30 mg	0.9000	30.000
	Mirtazapine	N06AX11	30 mg	Coated tablet	Blister 56 unit(s)	15 mg	0.8400	28.000
	Mirtazapine	N06AX11	30 mg	Coated tablet	Blister 56 unit(s)	30 mg	1.6800	56.000
	Mirtazapine	N06AX11	30 mg	Film-coated tablet	Blister 10 unit(s)	15 mg	0.1500	5.000
	Mirtazapine	N06AX11	30 mg	Film-coated tablet	Blister 10 unit(s)	30 mg	0.3000	10.000
	Mirtazapine	N06AX11	30 mg	Film-coated tablet	Blister 14 unit(s)	15 mg	0.2100	7.000
	Mirtazapine	N06AX11	30 mg	Film-coated tablet	Blister 14 unit(s)	30 mg	0.4200	14.000
	Mirtazapine	N06AX11	30 mg	Film-coated tablet	Blister 15 unit(s)	15 mg	0.2250	7.500
	Mirtazapine	N06AX11	30 mg	Film-coated tablet	Blister 15 unit(s)	30 mg	0.4500	15.000
	Mirtazapine	N06AX11	30 mg	Film-coated tablet	Blister 20 unit(s)	15 mg	0.3000	10.000
	Mirtazapine	N06AX11	30 mg	Film-coated tablet	Blister 20 unit(s)	30 mg	0.6000	20.000
	Mirtazapine	N06AX11	30 mg	Film-coated tablet	Blister 28 unit(s)	15 mg	0.4200	14.000
	Mirtazapine	N06AX11	30 mg	Film-coated tablet	Blister 28 unit(s)	30 mg	0.8400	28.000
	Mirtazapine	N06AX11	30 mg	Film-coated tablet	Blister 30 unit(s)	15 mg	0.4500	15.000
	Mirtazapine	N06AX11	30 mg	Film-coated tablet	Blister 30 unit(s)	30 mg	0.9000	30.000
	Mirtazapine	N06AX11	30 mg	Film-coated tablet	Blister 56 unit(s)	15 mg	0.8400	28.000
	Mirtazapine	N06AX11	30 mg	Film-coated tablet	Blister 56 unit(s)	30 mg	1.6800	56.000
	Mirtazapine	N06AX11	30 mg	Film-coated tablet	Blister 60 unit(s)	15 mg	0.9000	30.000

Table S1 (*cont.*): Drug packages by class, active ingredient, administration route, packaging details, and dosage (DDDs per package).

Drug class	Drug name	ATC code	DDD	Mode	Package type	Dosage/unit	Total dosage (g)	DDD/package
**Antidepressant**	Mirtazapine	N06AX11	30 mg	Film-coated tablet	Blister 60 unit(s)	30 mg	1.8000	60.000
	Moclobemide	N06AG02	300 mg	Film-coated tablet	Blister 10 unit(s)	150 mg	1.5000	5.000
	Moclobemide	N06AG02	300 mg	Film-coated tablet	Blister 20 unit(s)	150 mg	3.0000	10.000
	Moclobemide	N06AG02	300 mg	Film-coated tablet	Blister 60 unit(s)	150 mg	9.0000	30.000
	Moclobemide	N06AG02	300 mg	Film-coated tablet	Blister 60 unit(s)	300 mg	18.0000	60.000
	Nortriptyline	N06AA10	75 mg	Coated tablet	Blister 20 unit(s)	25 mg	0.5000	6.667
	Nortriptyline	N06AA10	75 mg	Coated tablet	Blister 60 unit(s)	25 mg	1.5000	20.000
	Paroxetine	N06AB05	20 mg	Tablet	Blister 10 unit(s)	20 mg	0.2000	10.000
	Paroxetine	N06AB05	20 mg	Tablet	Blister 20 unit(s)	20 mg	0.4000	20.000
	Paroxetine	N06AB05	20 mg	Tablet	Blister 60 unit(s)	20 mg	1.2000	60.000
	Paroxetine	N06AB05	20 mg	Film-coated tablet	Blister 10 unit(s)	20 mg	0.2000	10.000
	Paroxetine	N06AB05	20 mg	Film-coated tablet	Blister 20 unit(s)	20 mg	0.4000	20.000
	Paroxetine	N06AB05	20 mg	Film-coated tablet	Blister 30 unit(s)	20 mg	0.6000	30.000
	Paroxetine	N06AB05	20 mg	Film-coated tablet	Blister 60 unit(s)	20 mg	1.2000	60.000
	Paroxetine	N06AB05	20 mg	Film-coated tablet	Bottle 10 unit(s)	20 mg	0.2000	10.000
	Paroxetine	N06AB05	20 mg	Film-coated tablet	Bottle 30 unit(s)	20 mg	0.6000	30.000
	Paroxetine	N06AB05	20 mg	Film-coated tablet	Bottle 60 unit(s)	20 mg	1.2000	60.000
	Paroxetine	N06AB05	20 mg	Oral drops	Bottle 1x 30 ml	10 mg/ml	0.3000	15.000
	Pirlindole	-	200 mg	Tablet	Blister 60 unit(s)	100 mg	6.0000	30.000
	Pirlindole	-	200 mg	Tablet	Blister 60 unit(s)	75 mg	4.5000	22.500
	Pirlindole	-	200 mg	Tablet	Bottle 20 unit(s)	50 mg	1.0000	5.000
	Pirlindole	-	200 mg	Tablet	Bottle 60 unit(s)	50 mg	3.0000	15.000
	Reboxetine	N06AX18	8 mg	Tablet	Blister 20 unit(s)	4 mg	0.0800	10.000
	Reboxetine	N06AX18	8 mg	Tablet	Blister 60 unit(s)	4 mg	0.2400	30.000
	Sertraline	N06AB06	50 mg	Film-coated tablet	Blister 10 unit(s)	50 mg	0.5000	10.000
	Sertraline	N06AB06	50 mg	Film-coated tablet	Blister 14 unit(s)	50 mg	0.7000	14.000
	Sertraline	N06AB06	50 mg	Film-coated tablet	Blister 20 unit(s)	50 mg	1.0000	20.000
	Sertraline	N06AB06	50 mg	Film-coated tablet	Blister 30 unit(s)	100 mg	3.0000	60.000
	Sertraline	N06AB06	50 mg	Film-coated tablet	Blister 30 unit(s)	50 mg	1.5000	30.000
	Sertraline	N06AB06	50 mg	Film-coated tablet	Blister 56 unit(s)	100 mg	5.6000	112.000
	Sertraline	N06AB06	50 mg	Film-coated tablet	Blister 56 unit(s)	50 mg	2.8000	56.000
	Sertraline	N06AB06	50 mg	Film-coated tablet	Blister 60 unit(s)	100 mg	6.0000	120.000
	Sertraline	N06AB06	50 mg	Film-coated tablet	Blister 60 unit(s)	150 mg	9.0000	180.000
	Sertraline	N06AB06	50 mg	Film-coated tablet	Blister 60 unit(s)	200 mg	12.0000	240.000
	Sertraline	N06AB06	50 mg	Film-coated tablet	Blister 60 unit(s)	50 mg	3.0000	60.000
	Sertraline	N06AB06	50 mg	Oral solution concentrate	Bottle 1x 60 ml	20 mg/ml	1.2000	24.000
	Trazodone	N06AX05	300 mg	Tablet	Blister 20 unit(s)	100 mg	2.0000	6.667
	Trazodone	N06AX05	300 mg	Tablet	Blister 20 unit(s)	150 mg	3.0000	10.000
	Trazodone	N06AX05	300 mg	Tablet	Blister 60 unit(s)	100 mg	6.0000	20.000
	Trazodone	N06AX05	300 mg	Tablet	Blister 60 unit(s)	150 mg	9.0000	30.000
	Trazodone	N06AX05	300 mg	Modified-release tablet	Blister 20 unit(s)	150 mg	3.0000	10.000
	Trazodone	N06AX05	300 mg	Modified-release tablet	Blister 60 unit(s)	150 mg	9.0000	30.000
	Trazodone	N06AX05	300 mg	Extended-release tablet	Blister 10 unit(s)	150 mg	1.5000	5.000
	Trazodone	N06AX05	300 mg	Extended-release tablet	Blister 30 unit(s)	300 mg	9.0000	30.000
	Trazodone	N06AX05	300 mg	Film-coated tablet	Blister 20 unit(s)	100 mg	2.0000	6.667
	Trazodone	N06AX05	300 mg	Film-coated tablet	Blister 60 unit(s)	100 mg	6.0000	20.000
	Trimipramine	N06AA06	150 mg	Tablet	Blister 10 unit(s)	25 mg	0.2500	1.667
	Trimipramine	N06AA06	150 mg	Tablet	Blister 60 unit(s)	25 mg	1.5000	10.000
	Trimipramine	N06AA06	150 mg	Coated tablet	Blister 60 unit(s)	100 mg	6.0000	40.000
	Venlafaxine	N06AX16	100 mg	Extensed-release capsule	Blister 10 unit(s)	37.5 mg	0.3750	3.750
	Venlafaxine	N06AX16	100 mg	Extensed-release capsule	Blister 10 unit(s)	75 mg	0.7500	7.500
	Venlafaxine	N06AX16	100 mg	Extensed-release capsule	Blister 20 unit(s)	37.5 mg	0.7500	7.500
	Venlafaxine	N06AX16	100 mg	Extensed-release capsule	Blister 30 unit(s)	150 mg	4.5000	45.000
	Venlafaxine	N06AX16	100 mg	Extensed-release capsule	Blister 30 unit(s)	225 mg	6.7500	67.500
	Venlafaxine	N06AX16	100 mg	Extensed-release capsule	Blister 30 unit(s)	37.5 mg	1.1250	11.250
	Venlafaxine	N06AX16	100 mg	Extensed-release capsule	Blister 30 unit(s)	75 mg	2.2500	22.500
	Venlafaxine	N06AX16	100 mg	Extended-release tablet	Blister 10 unit(s)	37.5 mg	0.3750	3.750
	Venlafaxine	N06AX16	100 mg	Extended-release tablet	Blister 10 unit(s)	75 mg	0.7500	7.500
	Venlafaxine	N06AX16	100 mg	Extended-release tablet	Blister 30 unit(s)	150 mg	4.5000	45.000
	Venlafaxine	N06AX16	100 mg	Extended-release tablet	Blister 30 unit(s)	225 mg	6.7500	67.500
	Venlafaxine	N06AX16	100 mg	Extended-release tablet	Blister 30 unit(s)	37.5 mg	1.1250	11.250
	Venlafaxine	N06AX16	100 mg	Extended-release tablet	Blister 30 unit(s)	75 mg	2.2500	22.500
	Venlafaxine	N06AX16	100 mg	Film-coated tablet	Blister 10 unit(s)	37.5 mg	0.3750	3.750
	Venlafaxine	N06AX16	100 mg	Film-coated tablet	Blister 60 unit(s)	37.5 mg	2.2500	22.500
	Venlafaxine	N06AX16	100 mg	Film-coated tablet	Blister 60 unit(s)	75 mg	4.5000	45.000
	Venlafaxine	N06AX16	100 mg	Oral solution	Bottle 1x 60 ml	75 mg/ml	4.5000	45.000
	Vortioxetine	N06AX26	10 mg	Film-coated tablet	Blister 14 unit(s)	5 mg	0.0700	7.000
	Vortioxetine	N06AX26	10 mg	Film-coated tablet	Blister 28 unit(s)	10 mg	0.2800	28.000
	Vortioxetine	N06AX26	10 mg	Film-coated tablet	Blister 28 unit(s)	15 mg	0.4200	42.000
	Vortioxetine	N06AX26	10 mg	Film-coated tablet	Blister 28 unit(s)	20 mg	0.5600	56.000
	Vortioxetine	N06AX26	10 mg	Film-coated tablet	Blister 28 unit(s)	5 mg	0.1400	14.000
	Vortioxetine	N06AX26	10 mg	Oral drops	Bottle 1x 15 ml	20 mg/ml	0.3000	30.000
	Amissulpride	N05AL05	0.4 g	Tablet	Blister 20 unit(s)	50 mg	1.0000	2.500
	Amissulpride	N05AL05	0.4 g	Tablet	Blister 60 unit(s)	200 mg	12.0000	30.000
	Amissulpride	N05AL05	0.4 g	Tablet	Blister 60 unit(s)	50 mg	3.0000	7.500
	Amissulpride	N05AL05	0.4 g	Oral solution	Ampoule 20 unit(s) 10 ml	50 mg/10 ml	1.0000	2.500
	Aripiprazole	N05AX12	15 mg	Tablet	Blister 14 unit(s)	10 mg	0.1400	9.333
	Aripiprazole	N05AX12	15 mg	Tablet	Blister 14 unit(s)	15 mg	0.2100	14.000
	Aripiprazole	N05AX12	15 mg	Tablet	Blister 14 unit(s)	20 mg	0.2800	18.667
	Aripiprazole	N05AX12	15 mg	Tablet	Blister 14 unit(s)	30 mg	0.4200	28.000
	Aripiprazole	N05AX12	15 mg	Tablet	Blister 14 unit(s)	5 mg	0.0700	4.667
	Aripiprazole	N05AX12	15 mg	Tablet	Blister 28 unit(s)	10 mg	0.2800	18.667
	Aripiprazole	N05AX12	15 mg	Tablet	Blister 28 unit(s)	15 mg	0.4200	28.000
	Aripiprazole	N05AX12	15 mg	Tablet	Blister 28 unit(s)	20 mg	0.5600	37.333
	Aripiprazole	N05AX12	15 mg	Tablet	Blister 28 unit(s)	30 mg	0.8400	56.000
	Aripiprazole	N05AX12	15 mg	Tablet	Blister 28 unit(s)	5 mg	0.1400	9.333
	Aripiprazole	N05AX12	15 mg	Orodispersible tablet	Blister 28 unit(s)	10 mg	0.2800	18.667
	Aripiprazole	N05AX12	15 mg	Orodispersible tablet	Blister 28 unit(s)	15 mg	0.4200	28.000
	Aripiprazole	N05AX12	15 mg	Extended-release susp. for injection	Vial 1x 1.9 ml	400 mg	0.0000	30.080
	Aripiprazole	N05AX12	15 mg	Injectable solution	Vial 1x 1.3 ml	7.5 mg/ml	0.0000	0.650
	Aripiprazole	N05AX12	15 mg	Oral solution	Bottle 1x 150 ml	1 mg/ml	0.1500	10.000
**Antipsychotic**	Cariprazine	N05AX15	3 mg	Capsule	Blister 14 unit(s)	1.5 mg	0.0210	7.000
	Cariprazine	N05AX15	3 mg	Capsule	Blister 14 unit(s)	3 mg	0.0420	14.000
	Cariprazine	N05AX15	3 mg	Capsule	Blister 28 unit(s)	1.5 mg	0.0420	14.000
	Cariprazine	N05AX15	3 mg	Capsule	Blister 28 unit(s)	3 mg	0.0840	28.000

Table S1 (*cont.*): Drug packages by class, active ingredient, administration route, packaging details, and dosage (DDDs per package).

Drug class	Drug name	ATC code	DDD	Mode	Package type	Dosage/unit	Total dosage (g)	DDD/package
**Antipsychotic**	Cariprazine	N05AX15	3 mg	Capsule	Blister 28 unit(s)	4.5 mg	0.1260	42.000
	Cariprazine	N05AX15	3 mg	Capsule	Blister 28 unit(s)	6 mg	0.1680	56.000
	Chlorpromazine	N05AA01	300 mg	Coated tablet	Blister 20 unit(s)	25 mg	0.5000	1.667
	Chlorpromazine	N05AA01	300 mg	Coated tablet	Blister 60 unit(s)	100 mg	6.0000	20.000
	Chlorpromazine	N05AA01	300 mg	Coated tablet	Blister 60 unit(s)	25 mg	1.5000	5.000
	Chlorpromazine	N05AA01	300 mg	Oral drops	Dropper bottle 1x 30 ml	40 mg/ml	1.2000	4.000
	Chlorpromazine	N05AA01	300 mg	Injectable solution	Ampoule 5 unit(s) 2 ml	50 mg/2 ml	0.2500	2.500
	Chlorpromazine	N05AA01	300 mg	Injectable solution	Ampoule 6 unit(s) 5 ml	25 mg/5 ml	0.1500	1.500
	Ciamemazine	N05AA06	100 mg	Coated tablet	Blister 20 unit(s)	100 mg	2.0000	20.000
	Ciamemazine	N05AA06	100 mg	Coated tablet	Blister 60 unit(s)	100 mg	6.0000	60.000
	Ciamemazine	N05AA06	100 mg	Oral drops	Bottle 1x 30 ml	40 mg/ml	1.2000	12.000
	Clozapine	N05AH02	300 mg	Tablet	Blister 14 unit(s)	25 mg	0.3500	1.167
	Clozapine	N05AH02	300 mg	Tablet	Blister 20 unit(s)	25 mg	0.5000	1.667
	Clozapine	N05AH02	300 mg	Tablet	Blister 56 unit(s)	25 mg	1.4000	4.667
	Clozapine	N05AH02	300 mg	Tablet	Blister 60 unit(s)	100 mg	6.0000	20.000
	Clozapine	N05AH02	300 mg	Tablet	Blister 60 unit(s)	25 mg	1.5000	5.000
	Flufenazine	N05AB02	1 mg	Injectable solution	Ampoule 1x 1 ml	25 mg/ml	0.0250	25.000
	Flupentixol	N05AF01	4 mg	Injectable solution	Ampoule 1x 1 ml	100 mg/1 ml	0.1000	25.000
	Flupentixol	N05AF01	4 mg	Injectable solution	Ampoule 5 unit(s) 1 ml	20 mg/1 ml	0.1000	25.000
	Haloperidol	N05AD01	8 mg	Tablet	Blister 20 unit(s)	1 mg	0.0200	2.500
	Haloperidol	N05AD01	8 mg	Tablet	Blister 60 unit(s)	10 mg	0.6000	75.000
	Haloperidol	N05AD01	8 mg	Tablet	Blister 60 unit(s)	1 mg	0.0600	7.500
	Haloperidol	N05AD01	8 mg	Tablet	Blister 60 unit(s)	2 mg	0.1200	15.000
	Haloperidol	N05AD01	8 mg	Tablet	Blister 60 unit(s)	5 mg	0.3000	37.500
	Haloperidol	N05AD01	8 mg	Injectable solution	Ampoule 1x 1 ml	100 mg/1 ml	0.1000	30.300
	Haloperidol	N05AD01	8 mg	Injectable solution	Ampoule 1x 1 ml	50 mg/1 ml	0.0500	15.150
	Haloperidol	N05AD01	8 mg	Injectable solution	Ampoule 5 unit(s) 1 ml	5 mg/1 ml	0.0250	3.125
	Haloperidol	N05AD01	8 mg	Oral solution	Bottle 1x 30 ml	2 mg/ml	0.0600	7.500
	Levomepromazine	N05AA02	300 mg	Coated tablet	Blister 20 unit(s)	25 mg	0.5000	1.667
	Levomepromazine	N05AA02	300 mg	Coated tablet	Blister 60 unit(s)	100 mg	6.0000	20.000
	Levomepromazine	N05AA02	300 mg	Coated tablet	Blister 60 unit(s)	25 mg	1.5000	5.000
	Levomepromazine	N05AA02	300 mg	Oral drops	Bottle 1x 30 ml	40 mg/ml	1.2000	4.000
	Levomepromazine	N05AA02	300 mg	Injectable solution	Ampoule 6 unit(s) 1 ml	25 mg/1 ml	0.1500	1.500
	Melperone	N05AD03	300 mg	Film-coated tablet	Bottle 20 unit(s)	25 mg	0.5000	1.667
	Melperone	N05AD03	300 mg	Film-coated tablet	Bottle 60 unit(s)	25 mg	1.5000	5.000
	Melperone	N05AD03	300 mg	Film-coated tablet	Bottle 60 unit(s)	50 mg	3.0000	10.000
	Olanzapine	N05AH03	10 mg	Tablet	Blister 14 unit(s)	2.5 mg	0.0350	3.500
	Olanzapine	N05AH03	10 mg	Tablet	Blister 28 unit(s)	10 mg	0.2800	28.000
	Olanzapine	N05AH03	10 mg	Tablet	Blister 28 unit(s)	15 mg	0.4200	42.000
	Olanzapine	N05AH03	10 mg	Tablet	Blister 28 unit(s)	2.5 mg	0.0700	7.000
	Olanzapine	N05AH03	10 mg	Tablet	Blister 28 unit(s)	5 mg	0.1400	14.000
	Olanzapine	N05AH03	10 mg	Tablet	Blister 28 unit(s)	7.5 mg	0.2100	21.000
	Olanzapine	N05AH03	10 mg	Orodispersible tablet	Blister 28 unit(s)	10 mg	0.2800	28.000
	Olanzapine	N05AH03	10 mg	Orodispersible tablet	Blister 28 unit(s)	15 mg	0.4200	42.000
	Olanzapine	N05AH03	10 mg	Orodispersible tablet	Blister 28 unit(s)	20 mg	0.5600	56.000
	Olanzapine	N05AH03	10 mg	Orodispersible tablet	Blister 28 unit(s)	5 mg	0.1400	14.000
	Olanzapine	N05AH03	10 mg	Coated tablet	Blister 28 unit(s)	10 mg	0.2800	28.000
	Olanzapine	N05AH03	10 mg	Coated tablet	Blister 28 unit(s)	15 mg	0.4200	42.000
	Olanzapine	N05AH03	10 mg	Coated tablet	Blister 28 unit(s)	2.5 mg	0.0700	7.000
	Olanzapine	N05AH03	10 mg	Coated tablet	Blister 28 unit(s)	5 mg	0.1400	14.000
	Olanzapine	N05AH03	10 mg	Coated tablet	Blister 28 unit(s)	7.5 mg	0.2100	21.000
	Olanzapine	N05AH03	10 mg	Film-coated tablet	Blister 14 unit(s)	5 mg	0.0700	7.000
	Olanzapine	N05AH03	10 mg	Film-coated tablet	Blister 28 unit(s)	10 mg	0.2800	28.000
	Olanzapine	N05AH03	10 mg	Film-coated tablet	Blister 28 unit(s)	15 mg	0.4200	42.000
	Olanzapine	N05AH03	10 mg	Film-coated tablet	Blister 28 unit(s)	2.5 mg	0.0700	7.000
	Olanzapine	N05AH03	10 mg	Film-coated tablet	Blister 28 unit(s)	5 mg	0.1400	14.000
	Olanzapine	N05AH03	10 mg	Film-coated tablet	Blister 28 unit(s)	7.5 mg	0.2100	21.000
	Paliperidone	N05AX13	2.5 mg	Extended-release tablet	Blister 14 unit(s)	3 mg	0.0420	7.000
	Paliperidone	N05AX13	2.5 mg	Extended-release tablet	Blister 28 unit(s)	3 mg	0.0840	14.000
	Paliperidone	N05AX13	2.5 mg	Extended-release tablet	Blister 28 unit(s)	6 mg	0.1680	28.000
	Paliperidone	N05AX13	2.5 mg	Extended-release tablet	Blister 28 unit(s)	9 mg	0.2520	42.000
	Paliperidone	N05AX13	2.5 mg	Extended-release susp. for injection	Pre-filled syringe 1x	100 mg	0.1000	40.000
	Paliperidone	N05AX13	2.5 mg	Extended-release susp. for injection	Pre-filled syringe 1x	150 mg	0.1500	60.000
	Paliperidone	N05AX13	2.5 mg	Extended-release susp. for injection	Pre-filled syringe 1x	50 mg	0.0500	20.000
	Paliperidone	N05AX13	2.5 mg	Extended-release susp. for injection	Pre-filled syringe 1x	75 mg	0.0750	30.000
	Paliperidone	N05AX13	2.5 mg	Extended-release susp. for injection	Pre-filled syringe 1x 0.75 ml	75 mg	0.0750	30.000
	Paliperidone	N05AX13	2.5 mg	Extended-release susp. for injection	Pre-filled syringe 1x 0.875 ml	175 mg	0.0000	70.000
	Paliperidone	N05AX13	2.5 mg	Extended-release susp. for injection	Pre-filled syringe 1x 1.315 ml	263 mg	0.0000	105.200
	Paliperidone	N05AX13	2.5 mg	Extended-release susp. for injection	Pre-filled syringe 1x 1.75 ml	350 mg	0.0000	140.000
	Paliperidone	N05AX13	2.5 mg	Extended-release susp. for injection	Pre-filled syringe 1x 1 ml	100 mg	0.1000	40.000
	Paliperidone	N05AX13	2.5 mg	Extended-release susp. for injection	Pre-filled syringe 1x 2.625 ml	525 mg	0.0000	210.000
	Pimozide	N05AG02	4 mg	Tablet	Blister 20 unit(s)	4 mg	0.0800	20.000
	Pimozide	N05AG02	4 mg	Tablet	Blister 60 unit(s)	4 mg	0.2400	60.000
	Quetiapine	N05AH04	400 mg	Extended-release tablet	Blister 10 unit(s)	200 mg	2.0000	5.000
	Quetiapine	N05AH04	400 mg	Extended-release tablet	Blister 10 unit(s)	300 mg	3.0000	7.500
	Quetiapine	N05AH04	400 mg	Extended-release tablet	Blister 10 unit(s)	50 mg	0.5000	1.250
	Quetiapine	N05AH04	400 mg	Extended-release tablet	Blister 30 unit(s)	150 mg	4.5000	11.250
	Quetiapine	N05AH04	400 mg	Extended-release tablet	Blister 60 unit(s)	200 mg	12.0000	30.000
	Quetiapine	N05AH04	400 mg	Extended-release tablet	Blister 60 unit(s)	300 mg	18.0000	45.000
	Quetiapine	N05AH04	400 mg	Extended-release tablet	Blister 60 unit(s)	400 mg	24.0000	60.000
	Quetiapine	N05AH04	400 mg	Extended-release tablet	Blister 60 unit(s)	50 mg	3.0000	7.500
	Quetiapine	N05AH04	400 mg	Extended-release tablet	Bottle 10 unit(s)	200 mg	2.0000	5.000
	Quetiapine	N05AH04	400 mg	Film-coated tablet	Blister 10 unit(s)	25 mg	0.2500	0.625
	Quetiapine	N05AH04	400 mg	Film-coated tablet	Blister 10 unit(s)	25+100+200mg	0.6500	1.625
	Quetiapine	N05AH04	400 mg	Film-coated tablet	Blister 20 unit(s)	25 mg	0.5000	1.250
	Quetiapine	N05AH04	400 mg	Film-coated tablet	Blister 60 unit(s)	100 mg	6.0000	15.000
	Quetiapine	N05AH04	400 mg	Film-coated tablet	Blister 60 unit(s)	150 mg	9.0000	22.500
	Quetiapine	N05AH04	400 mg	Film-coated tablet	Blister 60 unit(s)	200 mg	12.0000	30.000
	Quetiapine	N05AH04	400 mg	Film-coated tablet	Blister 60 unit(s)	300 mg	18.0000	45.000
	Quetiapine	N05AH04	400 mg	Film-coated tablet	Blister 60 unit(s)	400 mg	24.0000	60.000
	Quetiapine	N05AH04	400 mg	Film-coated tablet	Blister 60 unit(s)	50 mg	3.0000	7.500
	Quetiapine	N05AH04	400 mg	Film-coated tablet	Blister 6 unit(s)	25 mg	0.1500	0.375
	Risperidone	N05AX08	5 mg	Orodispersible tablet	Blister 14 unit(s)	0.5 mg	0.0070	1.400
	Risperidone	N05AX08	5 mg	Orodispersible tablet	Blister 14 unit(s)	2 mg	0.0280	5.600
	Risperidone	N05AX08	5 mg	Orodispersible tablet	Blister 20 unit(s)	0.5 mg	0.0100	2.000

Table S1 (*cont.*): Drug packages by class, active ingredient, administration route, packaging details, and dosage (DDDs per package).

Drug class	Drug name	ATC code	DDD	Mode	Package type	Dosage/unit	Total dosage (g)	DDD/package
**Antipsychotic**	Risperidone	N05AX08	5 mg	Orodispersible tablet	Blister 20 unit(s)	1 mg	0.0200	4.000
	Risperidone	N05AX08	5 mg	Orodispersible tablet	Blister 28 unit(s)	2 mg	0.0560	11.200
	Risperidone	N05AX08	5 mg	Orodispersible tablet	Blister 56 unit(s)	0.5 mg	0.0280	5.600
	Risperidone	N05AX08	5 mg	Orodispersible tablet	Blister 56 unit(s)	1 mg	0.0560	11.200
	Risperidone	N05AX08	5 mg	Orodispersible tablet	Blister 56 unit(s)	2 mg	0.1120	22.400
	Risperidone	N05AX08	5 mg	Orodispersible tablet	Blister 56 unit(s)	3 mg	0.1680	33.600
	Risperidone	N05AX08	5 mg	Orodispersible tablet	Blister 56 unit(s)	4 mg	0.2240	44.800
	Risperidone	N05AX08	5 mg	Orodispersible tablet	Blister 60 unit(s)	0.5 mg	0.0300	6.000
	Risperidone	N05AX08	5 mg	Orodispersible tablet	Blister 60 unit(s)	1 mg	0.0600	12.000
	Risperidone	N05AX08	5 mg	Orodispersible tablet	Blister 60 unit(s)	2 mg	0.1200	24.000
	Risperidone	N05AX08	5 mg	Film-coated tablet	Blister 20 unit(s)	0.5 mg	0.0100	2.000
	Risperidone	N05AX08	5 mg	Film-coated tablet	Blister 20 unit(s)	1 mg	0.0200	4.000
	Risperidone	N05AX08	5 mg	Film-coated tablet	Blister 60 unit(s)	0.5 mg	0.0300	6.000
	Risperidone	N05AX08	5 mg	Film-coated tablet	Blister 60 unit(s)	1 mg	0.0600	12.000
	Risperidone	N05AX08	5 mg	Film-coated tablet	Blister 60 unit(s)	2 mg	0.1200	24.000
	Risperidone	N05AX08	5 mg	Film-coated tablet	Blister 60 unit(s)	3 mg	0.1800	36.000
	Risperidone	N05AX08	5 mg	Film-coated tablet	Blister 60 unit(s)	4 mg	0.2400	48.000
	Risperidone	N05AX08	5 mg	Suspension for injection	Vial 1x 2 ml	25 mg/2 ml	0.0250	9.259
	Risperidone	N05AX08	5 mg	Suspension for injection	Vial 1x 2 ml	37.5 mg/2 ml	0.0375	13.889
	Risperidone	N05AX08	5 mg	Suspension for injection	Vial 1x 2 ml	50 mg/2 ml	0.0500	18.519
	Risperidone	N05AX08	5 mg	Extended-release susp. for injection	Vial 1x 2 ml	25 mg/2 ml	0.0250	9.259
	Risperidone	N05AX08	5 mg	Extended-release susp. for injection	Vial 1x 2 ml	37.5 mg/2 ml	0.0375	13.889
	Risperidone	N05AX08	5 mg	Extended-release susp. for injection	Vial 1x 2 ml	50 mg/2 ml	0.0500	18.519
	Risperidone	N05AX08	5 mg	Extended-release susp. for injection	Pre-filled syringe 1x 0.383 ml	75 mg/0.383 ml	0.0750	27.777
	Risperidone	N05AX08	5 mg	Extended-release susp. for injection	Pre-filled syringe 1x 0.49 ml	100 mg/0.490 ml	0.1000	37.037
	Risperidone	N05AX08	5 mg	Oral solution	Bottle 1x 30 ml	1 mg/ml	0.0300	6.000
	Sulpiride	N05AL01	800 mg	Capsule	Blister 20 unit(s)	50 mg	1.0000	1.250
	Sulpiride	N05AL01	800 mg	Capsule	Blister 60 unit(s)	50 mg	3.0000	3.750
	Sulpiride	N05AL01	800 mg	Tablet	Blister 60 unit(s)	200 mg	12.0000	15.000
	Tiapride	N05AL03	400 mg	Tablet	Blister 20 unit(s)	100 mg	2.0000	5.000
	Tiapride	N05AL03	400 mg	Tablet	Blister 60 unit(s)	100 mg	6.0000	15.000
	Tiapride	N05AL03	400 mg	Injectable solution	Ampoule 6 unit(s) 2 ml	100 mg/2 ml	0.6000	1.500
	Ziprasidone	N05AE04	80 mg	Capsule	Blister 14 unit(s)	20 mg	0.2800	3.500
	Ziprasidone	N05AE04	80 mg	Capsule	Blister 14 unit(s)	40 mg	0.5600	7.000
	Ziprasidone	N05AE04	80 mg	Capsule	Blister 56 unit(s)	20 mg	1.1200	14.000
	Ziprasidone	N05AE04	80 mg	Capsule	Blister 56 unit(s)	40 mg	2.2400	28.000
	Ziprasidone	N05AE04	80 mg	Capsule	Blister 56 unit(s)	60 mg	3.3600	42.000
	Ziprasidone	N05AE04	80 mg	Capsule	Blister 56 unit(s)	80 mg	4.4800	56.000
	Zotepine	N05AX11	200 mg	Coated tablet	Blister 20 unit(s)	25 mg	0.5000	2.500
	Zotepine	N05AX11	200 mg	Coated tablet	Blister 60 unit(s)	100 mg	6.0000	30.000
	Zotepine	N05AX11	200 mg	Coated tablet	Blister 60 unit(s)	25 mg	1.5000	7.500
	Zotepine	N05AX11	200 mg	Coated tablet	Blister 60 unit(s)	50 mg	3.0000	15.000
	Zuclopenthixol	N05AF05	30 mg	Injectable solution	Ampoule 1x 1 ml	200 mg/1 ml	0.2000	6.667
	Zuclopenthixol	N05AF05	30 mg	Injectable solution	Ampoule 1x 1 ml	50 mg/1 ml	0.0500	1.667
**Benzodiazepine or similar**	Alprazolam	N05BA12	1 mg	Tablet	Blister 20 unit(s)	0.25 mg	0.0050	5.000
	Alprazolam	N05BA12	1 mg	Tablet	Blister 20 unit(s)	0.5 mg	0.0100	10.000
	Alprazolam	N05BA12	1 mg	Tablet	Blister 20 unit(s)	1 mg	0.0200	20.000
	Alprazolam	N05BA12	1 mg	Tablet	Blister 60 unit(s)	0.25 mg	0.0150	15.000
	Alprazolam	N05BA12	1 mg	Tablet	Blister 60 unit(s)	0.5 mg	0.0300	30.000
	Alprazolam	N05BA12	1 mg	Tablet	Blister 60 unit(s)	1 mg	0.0600	60.000
	Alprazolam	N05BA12	1 mg	Modified-release tablet	Blister 20 unit(s)	0.5 mg	0.0100	10.000
	Alprazolam	N05BA12	1 mg	Modified-release tablet	Blister 20 unit(s)	1 mg	0.0200	20.000
	Alprazolam	N05BA12	1 mg	Modified-release tablet	Blister 20 unit(s)	2 mg	0.0400	40.000
	Alprazolam	N05BA12	1 mg	Modified-release tablet	Blister 20 unit(s)	3 mg	0.0600	60.000
	Alprazolam	N05BA12	1 mg	Modified-release tablet	Blister 40 unit(s)	0.5 mg	0.0200	20.000
	Alprazolam	N05BA12	1 mg	Modified-release tablet	Blister 40 unit(s)	1 mg	0.0400	40.000
	Alprazolam	N05BA12	1 mg	Modified-release tablet	Blister 60 unit(s)	0.5 mg	0.0300	30.000
	Alprazolam	N05BA12	1 mg	Modified-release tablet	Blister 60 unit(s)	1 mg	0.0600	60.000
	Alprazolam	N05BA12	1 mg	Modified-release tablet	Blister 60 unit(s)	2 mg	0.1200	120.000
	Alprazolam	N05BA12	1 mg	Modified-release tablet	Blister 60 unit(s)	3 mg	0.1800	180.000
	Alprazolam	N05BA12	1 mg	Sublingual tablet	Blister 10 unit(s)	0.5 mg	0.0050	5.000
	Alprazolam	N05BA12	1 mg	Sublingual tablet	Blister 20 unit(s)	0.5 mg	0.0100	10.000
	Alprazolam	N05BA12	1 mg	Sublingual tablet	Blister 60 unit(s)	0.5 mg	0.0300	30.000
	Alprazolam	N05BA12	1 mg	Sublingual tablet	Blister 60 unit(s)	1 mg	0.0600	60.000
	Bromazepam	N05BA08	10 mg	Capsule	Blister 20 unit(s)	1.5 mg	0.0300	3.000
	Bromazepam	N05BA08	10 mg	Capsule	Blister 20 unit(s)	3 mg	0.0600	6.000
	Bromazepam	N05BA08	10 mg	Capsule	Blister 60 unit(s)	1.5 mg	0.0900	9.000
	Bromazepam	N05BA08	10 mg	Capsule	Blister 60 unit(s)	3 mg	0.1800	18.000
	Bromazepam	N05BA08	10 mg	Tablet	Blister 20 unit(s)	1.5 mg	0.0300	3.000
	Bromazepam	N05BA08	10 mg	Tablet	Blister 20 unit(s)	3 mg	0.0600	6.000
	Bromazepam	N05BA08	10 mg	Tablet	Blister 20 unit(s)	6 mg	0.1200	12.000
	Bromazepam	N05BA08	10 mg	Tablet	Blister 40 unit(s)	1.5 mg	0.0600	6.000
	Bromazepam	N05BA08	10 mg	Tablet	Blister 40 unit(s)	3 mg	0.1200	12.000
	Bromazepam	N05BA08	10 mg	Tablet	Blister 40 unit(s)	6 mg	0.2400	24.000
	Bromazepam	N05BA08	10 mg	Tablet	Blister 60 unit(s)	1.5 mg	0.0900	9.000
	Bromazepam	N05BA08	10 mg	Tablet	Blister 60 unit(s)	3 mg	0.1800	18.000
	Bromazepam	N05BA08	10 mg	Tablet	Blister 60 unit(s)	6 mg	0.3600	36.000
	Brotizolam	N05CD09	0.25 mg	Tablet	Blister 14 unit(s)	0.25 mg	0.0035	14.000
	Cetazolam	N05BA10	30 mg	Capsule	Bottle 20 unit(s)	15 mg	0.3000	10.000
	Cetazolam	N05BA10	30 mg	Capsule	Bottle 20 unit(s)	30 mg	0.6000	20.000
	Cetazolam	N05BA10	30 mg	Capsule	Bottle 60 unit(s)	15 mg	0.9000	30.000
	Cetazolam	N05BA10	30 mg	Capsule	Bottle 60 unit(s)	30 mg	1.8000	60.000
	Clobazam	N05BA09	20 mg	Tablet	Blister 20 unit(s)	10 mg	0.2000	10.000
	Clobazam	N05BA09	20 mg	Tablet	Blister 20 unit(s)	20 mg	0.4000	20.000
	Clobazam	N05BA09	20 mg	Tablet	Blister 30 unit(s)	10 mg	0.3000	15.000
	Clobazam	N05BA09	20 mg	Tablet	Blister 30 unit(s)	20 mg	0.6000	30.000
	Clorazepate dip.	N05BA05	20 mg	Capsule	Blister 20 unit(s)	10 mg	0.2000	10.000
	Clorazepate dip.	N05BA05	20 mg	Capsule	Blister 20 unit(s)	15 mg	0.3000	15.000
	Clorazepate dip.	N05BA05	20 mg	Capsule	Blister 20 unit(s)	5 mg	0.1000	5.000
	Clorazepate dip.	N05BA05	20 mg	Capsule	Blister 60 unit(s)	10 mg	0.6000	30.000
	Clorazepate dip.	N05BA05	20 mg	Capsule	Blister 60 unit(s)	15 mg	0.9000	45.000
	Clorazepate dip.	N05BA05	20 mg	Capsule	Blister 60 unit(s)	5 mg	0.3000	15.000
	Chlordiazepoxide	N05BA02	30 mg	Coated tablet	Blister 20 unit(s)	5 mg	0.1000	3.333
	Chlordiazepoxide	N05BA02	30 mg	Coated tablet	Blister 60 unit(s)	10 mg	0.6000	20.000
	Chlordiazepoxide	N05BA02	30 mg	Coated tablet	Blister 60 unit(s)	5 mg	0.3000	10.000

Table S1 (*cont.*): Drug packages by class, active ingredient, administration route, packaging details, and dosage (DDDs per package).

Drug class	Drug name	ATC code	DDD	Mode	Package type	Dosage/unit	Total dosage (g)	DDD/package
**Benzodiazepine or similar**	Chlordiazepoxide + clid. brom.	A03CA02	30 mg	Coated tablet	Blister 20 unit(s)	5 mg + 2.5 mg	0.1000	3.333
	Chlordiazepoxide + clid. brom.	A03CA02	30 mg	Coated tablet	Blister 60 unit(s)	5 mg + 2.5 mg	0.3000	10.000
	Cloxazolam	N05BA22	6 mg	Tablet	Blister 20 unit(s)	1 mg	0.0200	3.333
	Cloxazolam	N05BA22	6 mg	Tablet	Blister 20 unit(s)	2 mg	0.0400	6.667
	Cloxazolam	N05BA22	6 mg	Tablet	Blister 60 unit(s)	1 mg	0.0600	10.000
	Cloxazolam	N05BA22	6 mg	Tablet	Blister 60 unit(s)	2 mg	0.1200	20.000
	Diazepam	N05BA01	10 mg	Capsule	Blister 20 unit(s)	3 mg	0.0600	6.000
	Diazepam	N05BA01	10 mg	Capsule	Blister 20 unit(s)	6 mg	0.1200	12.000
	Diazepam	N05BA01	10 mg	Capsule	Blister 60 unit(s)	3 mg	0.1800	18.000
	Diazepam	N05BA01	10 mg	Capsule	Blister 60 unit(s)	6 mg	0.3600	36.000
	Diazepam	N05BA01	10 mg	Extensed-release capsule	Blister 20 unit(s)	10 mg	0.2000	20.000
	Diazepam	N05BA01	10 mg	Extensed-release capsule	Blister 60 unit(s)	10 mg	0.6000	60.000
	Diazepam	N05BA01	10 mg	Tablet	Blister 20 unit(s)	10 mg	0.2000	20.000
	Diazepam	N05BA01	10 mg	Tablet	Blister 20 unit(s)	5 mg	0.1000	10.000
	Diazepam	N05BA01	10 mg	Tablet	Blister 25 unit(s)	10 mg	0.2500	25.000
	Diazepam	N05BA01	10 mg	Tablet	Blister 25 unit(s)	5 mg	0.1250	12.500
	Diazepam	N05BA01	10 mg	Tablet	Blister 40 unit(s)	10 mg	0.4000	40.000
	Diazepam	N05BA01	10 mg	Tablet	Blister 40 unit(s)	5 mg	0.2000	20.000
	Diazepam	N05BA01	10 mg	Tablet	Blister 60 unit(s)	10 mg	0.6000	60.000
	Diazepam	N05BA01	10 mg	Tablet	Blister 60 unit(s)	5 mg	0.3000	30.000
	Diazepam	N05BA01	10 mg	Rectal solution	Cannula 5 unit(s) 2.5 ml	10 mg/2.5 ml	0.0500	5.000
	Diazepam	N05BA01	10 mg	Rectal solution	Cannula 5 unit(s) 2.5 ml	5 mg/2.5 ml	0.0250	2.500
	Diazepam	N05BA01	10 mg	Rectal solution	Unidosage 4 unit(s) 2.5 ml	5 mg/2.5 ml	0.0200	2.000
	Estazolam	N05CD04	3 mg	Tablet	Blister 14 unit(s)	2 mg	0.0280	9.333
	Estazolam	N05CD04	3 mg	Tablet	Blister 28 unit(s)	2 mg	0.0560	18.667
	Flurazepam	N05CD01	30 mg	Capsule	Blister 14 unit(s)	30 mg	0.4200	14.000
	Flurazepam	N05CD01	30 mg	Capsule	Blister 20 unit(s)	15 mg	0.3000	10.000
	Flurazepam	N05CD01	30 mg	Capsule	Blister 20 unit(s)	30 mg	0.6000	20.000
	Loprazolam	N05CD11	1 mg	Tablet	Blister 15 unit(s)	1 mg	0.0150	15.000
	Lorazepam	N05BA06	2.5 mg	Tablet	Blister 20 unit(s)	1 mg	0.0200	8.000
	Lorazepam	N05BA06	2.5 mg	Tablet	Blister 20 unit(s)	2.5 mg	0.0500	20.000
	Lorazepam	N05BA06	2.5 mg	Tablet	Blister 20 unit(s)	5 mg	0.1000	40.000
	Lorazepam	N05BA06	2.5 mg	Tablet	Blister 30 unit(s)	1 mg	0.0300	12.000
	Lorazepam	N05BA06	2.5 mg	Tablet	Blister 30 unit(s)	2.5 mg	0.0750	30.000
	Lorazepam	N05BA06	2.5 mg	Tablet	Blister 40 unit(s)	1 mg	0.0400	16.000
	Lorazepam	N05BA06	2.5 mg	Tablet	Blister 40 unit(s)	2.5 mg	0.1000	40.000
	Lorazepam	N05BA06	2.5 mg	Tablet	Blister 60 unit(s)	1 mg	0.0600	24.000
	Lorazepam	N05BA06	2.5 mg	Tablet	Blister 60 unit(s)	2.5 mg	0.1500	60.000
	Lorazepam	N05BA06	2.5 mg	Tablet	Blister 60 unit(s)	5 mg	0.3000	120.000
	Lorazepam	N05BA06	2.5 mg	Tablet	Bottle 20 unit(s)	5 mg	0.1000	40.000
	Lorazepam	N05BA06	2.5 mg	Tablet	Bottle 60 unit(s)	5 mg	0.3000	120.000
	Mexazolam	N05BA25	3 mg	Tablet	Blister 20 unit(s)	1 mg	0.0200	6.667
	Mexazolam	N05BA25	3 mg	Tablet	Blister 60 unit(s)	1 mg	0.0600	20.000
	Midazolam	N05CD08	15 mg	Coated tablet	Blister 14 unit(s)	15 mg	0.2100	14.000
	Midazolam	N05CD08	15 mg	Coated tablet	Blister 20 unit(s)	15 mg	0.3000	20.000
	Midazolam	N05CD08	15 mg	Buccal solution	Pre-filled syringe 4x 0.5 ml	2.5 mg/0.5 ml	0.0100	0.667
	Midazolam	N05CD08	15 mg	Buccal solution	Pre-filled syringe 4x 1.5 ml	7.5 mg/1.5 ml	0.0300	2.000
	Midazolam	N05CD08	15 mg	Buccal solution	Pre-filled syringe 4x 1 ml	5 mg/1 ml	0.0200	1.333
	Midazolam	N05CD08	15 mg	Buccal solution	Pre-filled syringe 4x 2 ml	10 mg/2 ml	0.0400	2.667
	Oxazepam	N05BA04	50 mg	Tablet	Blister 30 unit(s)	15 mg	0.4500	9.000
	Oxazepam	N05BA04	50 mg	Tablet	Blister 30 unit(s)	50 mg	1.5000	30.000
	Prazepam	N05BA11	30 mg	Tablet	Blister 20 unit(s)	10 mg	0.2000	6.667
	Prazepam	N05BA11	30 mg	Tablet	Blister 60 unit(s)	10 mg	0.6000	20.000
	Temazepam	N05CD07	20 mg	Soft capsule	Blister 14 unit(s)	20 mg	0.2800	14.000
	Triazolam	N05CD05	0.25 mg	Tablet	Blister 20 unit(s)	0.25 mg	0.0050	20.000
	Zolpidem	N05CF02	10 mg	Film-coated tablet	Blister 14 units	10 mg	0.1400	14.000
	Zolpidem	N05CF02	10 mg	Film-coated tablet	Blister 14 units	10 mg	0.1400	20.000
**Mood stabiliser**	Lamotrigine	N03AX09	300 mg	Tablet	Blister 14 units	25 mg	0.3500	1.167
	Lamotrigine	N03AX09	300 mg	Tablet	Blister 14 units	50 mg	0.7000	2.333
	Lamotrigine	N03AX09	300 mg	Tablet	Blister 21 units	25 mg	0.5250	1.750
	Lamotrigine	N03AX09	300 mg	Tablet	Blister 42 units	25 mg	1.0500	3.500
	Lamotrigine	N03AX09	300 mg	Tablet	Blister 42 units	50 mg	2.1000	7.000
	Lamotrigine	N03AX09	300 mg	Tablet	Blister 56 units	100 mg	5.6000	18.667
	Lamotrigine	N03AX09	300 mg	Tablet	Blister 56 units	200 mg	11.2000	37.333
	Lamotrigine	N03AX09	300 mg	Tablet	Blister 56 units	25 mg	1.4000	4.667
	Lamotrigine	N03AX09	300 mg	Tablet	Blister 56 units	50 mg	2.8000	9.333
	Lamotrigine	N03AX09	300 mg	Dispersible or chewable tablet	Blister 14 units	5 mg	0.0700	0.233
	Lamotrigine	N03AX09	300 mg	Dispersible or chewable tablet	Blister 56 units	25 mg	1.4000	4.667
	Lamotrigine	N03AX09	300 mg	Dispersible or chewable tablet	Bottle 14 units	5 mg	0.0700	0.233
	Lamotrigine	N03AX09	300 mg	Dispersible or chewable tablet	Bottle 30 units	2 mg	0.0600	0.200
	Lithium	N05AN01	24 mmol	Modified-release tablet	Blister 100 units	400 mg	40.0000	45.133
	Lithium	N05AN01	24 mmol	Modified-release tablet	Bottle 100 units	400 mg	40.0000	45.133
	Valproic acid	N03AG01	1500 mg	Modified-release tablet	Blister 20 units	300 mg	6.0000	4.000
	Valproic acid	N03AG01	1500 mg	Modified-release tablet	Blister 60 units	300 mg	18.0000	12.000
	Valproic acid	N03AG01	1500 mg	Modified-release tablet	Blister 60 units	500 mg	30.0000	20.000
	Valproic acid	N03AG01	1500 mg	Gastro-resistant capsule	Blister 60 units	500 mg	30.0000	20.000
	Valproic acid	N03AG01	1500 mg	Coated tablet	Blister 20 units	200 mg	4.0000	2.667
	Valproic acid	N03AG01	1500 mg	Coated tablet	Blister 60 units	200 mg	12.0000	8.000
	Valproic acid	N03AG01	1500 mg	Extended-release capsule	Tablet recipient 50 units	150 mg	7.5000	5.000
	Valproic acid	N03AG01	1500 mg	Extended-release capsule	Tablet recipient 50 units	300 mg	15.0000	10.000
	Valproic acid	N03AG01	1500 mg	Modified-release granules	Sachet 30 units	100 mg	3.0000	2.000
	Valproic acid	N03AG01	1500 mg	Modified-release granules	Sachet 30 units	1000 mg	30.0000	20.000
**Others**	Pregabalin	N02BF02	300 mg	Capsule	Blister 14 units	25 mg	0.3500	1.167
	Pregabalin	N02BF02	300 mg	Capsule	Blister 14 units	75 mg	1.0500	3.500
	Pregabalin	N02BF02	300 mg	Capsule	Blister 56 units	150 mg	8.4000	28.000
	Pregabalin	N02BF02	300 mg	Capsule	Blister 56 units	225 mg	12.6000	42.000
	Pregabalin	N02BF02	300 mg	Capsule	Blister 56 units	25 mg	1.4000	4.667
	Pregabalin	N02BF02	300 mg	Capsule	Blister 56 units	300 mg	16.8000	56.000
	Pregabalin	N02BF02	300 mg	Capsule	Blister 56 units	50 mg	2.8000	9.333
	Pregabalin	N02BF02	300 mg	Capsule	Blister 56 units	75 mg	4.2000	14.000
	Pregabalin	N02BF02	300 mg	Capsule	Blister 84 units	100 mg	5.6000	18.667
	Pregabalin	N02BF02	300 mg	Capsule	Blister 84 units	200 mg	11.2000	37.333
